# Recent Advances in Design Strategies and Multifunctionality of Flexible Electromagnetic Interference Shielding Materials

**DOI:** 10.1007/s40820-022-00823-7

**Published:** 2022-03-25

**Authors:** Junye Cheng, Chuanbing Li, Yingfei Xiong, Huibin Zhang, Hassan Raza, Sana Ullah, Jinyi Wu, Guangping Zheng, Qi Cao, Deqing Zhang, Qingbin Zheng, Renchao Che

**Affiliations:** 1 School of Science and Engineering, The Chinese University of Hong Kong, Shenzhen, 518172 People’s Republic of China; 2 School of Materials Science and Engineering, Qiqihar University, Qiqihar, 161006 People’s Republic of China; 3 Laboratory of Advanced Materials, Shanghai Key Lab of Molecular Catalysis and Innovative Materials, Department of Materials Science, Fudan University, Shanghai, 200438 People’s Republic of China; 4 Department of Mechanical Engineering, Hong Kong Polytechnic University, Hung Hom, Kowloon, Hong Kong People’s Republic of China; 5 Key Laboratory of Energy Thermal Conversion and Control of Ministry of Education, School of Energy and Environment, Southeast University, Nanjing, 210096 People’s Republic of China

**Keywords:** Flexible shielding materials, Green shielding index, Multifunctionalities, EMI shielding mechanism

## Abstract

With rapid development of 5G communication technologies, electromagnetic interference (EMI) shielding for electronic devices has become an urgent demand in recent years, where the development of corresponding EMI shielding materials against detrimental electromagnetic radiation plays an essential role. Meanwhile, the EMI shielding materials with high flexibility and functional integrity are highly demanded for emerging shielding applications. Hitherto, a variety of flexible EMI shielding materials with lightweight and multifunctionalities have been developed. In this review, we not only introduce the recent development of flexible EMI shielding materials, but also elaborate the EMI shielding mechanisms and the index for "green EMI shielding" performance. In addition, the construction strategies for sophisticated multifunctionalities of flexible shielding materials are summarized. Finally, we propose several possible research directions for flexible EMI shielding materials in near future, which could be inspirational to the fast-growing next-generation flexible electronic devices with reliable and multipurpose protections as offered by EMI shielding materials.

## Highlights

- Detailed summary of current trends in the advancement of flexible EMI shielding materials.
- The theoretical shielding mechanisms and the latest concept of "green shielding" index (g_s_) are outlined.
- Functional applications of flexible EMI shielding materials are introduced from thermal conductivity, hydrophobicity to transparency, sensing even multiple functions.
- Exclusive insights in challenges and future design strategies opportunities for flexible EMI shielding materials are provided.

## Graphical abstract

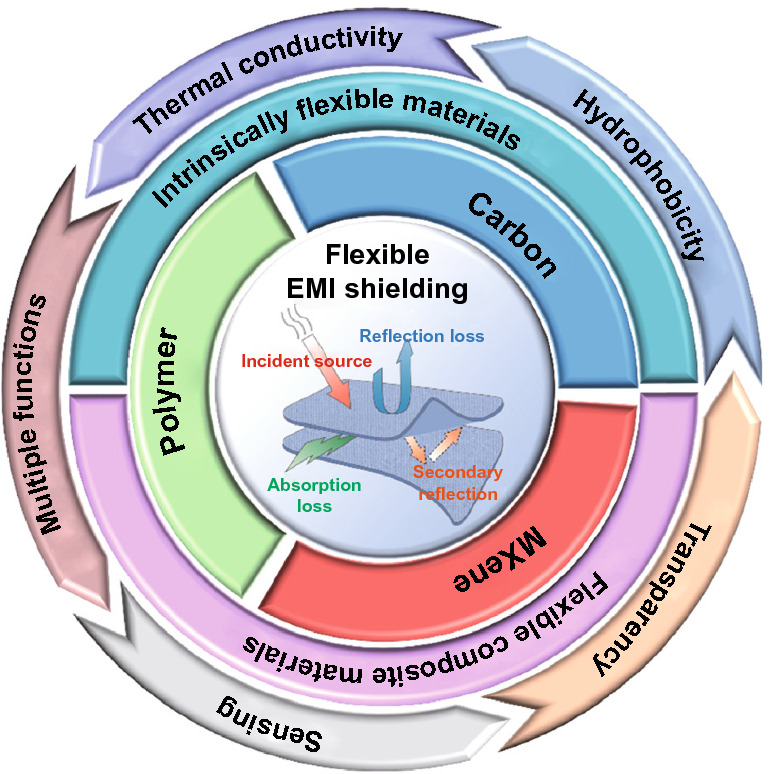

## Introduction

In modern society, electromagnetic radiation has become omnipresent in environment because of the tremendously growing usage of mobile phones, Wi-Fi and Bluetooth devices all around the world. It is reported that the number of wireless local area network (WLAN) connected devices in major cities worldwide has doubled from 2016 to 2021 [1], exposing the public to potential health risk that has yet to be adequately assessed. As early as in 2011, the French International Agency for Research on Cancer (IARC), which was authorized by World Health Organization (WHO), has classified that the electromagnetic radiation within 30 to 300 GHz could be carcinogenic [2]. It is known that 4G network and household appliances like microwave ovens mainly use the frequencies around 2.4 GHz. In recent years, a large number of reports have revealed the adverse effects of electromagnetic radiation in microwave frequencies around 2.4 GHz on the central nervous system of human beings, which could cause sleep disorders and wakefulness [3, 4], learning/memory impairment [5] and physical/cognitive abnormality [6]. In addition, the significantly increased incidences of malignant gliomas and schwannomas in male rats also turn out to be associated with the prolonged exposure to the electromagnetic radiation at 900 MHz to 1.8 GHz [7, 8].

Particularly, the public concern on the safety of electromagnetic radiation is further growing with the rapid advances in 5G technologies very recently. According to the 3rd Generation Partnership Project (3GPP) specification of TS 38.104 [9], the 5G-FR1 (sub-6 GHz) network could cover the 450 MHz to 6 GHz band, while mainly works in the n77 (3.3–4.2 GHz) and n79 (4.4–5.0 GHz) bands. Compared to traditional 4G networks which mainly work at around 2.4 GHz, it is obvious that the emerging 5G networks work at higher frequencies, and thus would emit electromagnetic radiation with higher energy. Therefore, such radiation can cause more serious health and safety issues to human bodies. Moreover, besides these possible health and safety issues to human beings, the electromagnetic radiation could also strongly interfere with the electronic devices [10] due to the interaction between electrons in the metallic conductor and the electric fields in the radiation, resulting in the malfunction of electronic devices [11, 12]. Therefore, the development of corresponding electromagnetic interference (EMI) shielding coatings, layers and devices that could resist harmful electromagnetic pollution is essential for the normally operation of electronic devices [13, 14] and the guarantee of human health and safety [15].

To develop high-performance and reliable EMI shielding devices, the EMI shielding materials play a fundamental role by absorbing or reflecting incident electromagnetic wave (EMW) to avoid it penetrating across the shielding layer [16, 17]. Generally, the EMWs are composed of magnetic fields and electric fields, which are perpendicular to each other. In this regard, EMI shielding mechanisms can be primarily categorized into electric shielding, magnetic shielding and electric–magnetic coupling EMI shielding [18]. According to the electromagnetic theory, the electric field and magnetic field of high-frequency EMWs that characterize the radiation strength are interdependent to each other; thus, the shielding of either of them can lead to the vanishing of the other. That is the main reason why traditional EMI shielding materials are mostly conductive materials. At present, the EMI shielding materials actually used in our daily life are mostly conductive materials [19]. According to the different requirements on varied occasions, it could be divided into conductive cloth, conductive rubbers, conductive adhesives and conductive coatings. Generally, conductive cloth are mostly used for flexible human-protective equipment; Conductive rubbers and adhesives are more used in electronic devices owing to their high processability and sealing ability, while the adhesives are usually more indispensable and stable. Conductive coatings have already been widely used in furniture such as chassis. However, as the advances in flexible electronic technology, the EMI shielding materials with the low density, high corrosion resistance, superior mechanical flexibility and low-processing-cost features are extremely desirable for practical applications [20].

Meanwhile, with the rapid advances in flexible and wearable electronics [21], the corresponding EMI shielding materials should also possess low density (*i.e.,* lightweight), high thermal stability, appreciable mechanical flexibility and corrosion resistance besides effective EMI shielding performance [22]. Currently, the developed flexible EMI shielding materials are mainly based on carbon materials [23, 24], polymers [25–27] and MXene-based materials [28]. Particularly, MXene materials, as a novel branch of two-dimensional (2D) inorganic materials, are generally known as transition metal carbides, nitrides or carbonitrides. Although MXenes contain metal elements, they have displayed many unique physicochemical properties which are favorable for flexible EMI shielding, while maintaining the metal-comparable high electrical conductivity at the same time. In the past decade, the number of publications related to flexible EMI shielding materials has been increasing quickly [11, 29, 30]. However, this important topic has been rarely summarized systematically in review articles till now.

This review specifically focuses on various flexible EMI shielding materials for advanced flexible electronic devices and equipment, such as intrinsically flexible substrate/matrix materials and composite flexible matrixes (e.g., carbon-based, MXene-based and polymer-based flexible composite materials). Further, we also analyze the EMI shielding mechanisms and the correlation between EMI shielding efficiencies and absorption/reflection components of EMW, based on which the different construction strategies for the shielding materials are illustrated. Furthermore, we also summarize the multifunctional integration of flexible EMI shielding materials toward extended application fields. Finally, current research challenges and prospective research directions are pointed out for future development of advanced flexible shielding materials.

## Electromagnetic Shielding and Attenuation Mechanisms

### Shielding Modes and Shielding Efficiency

**Fig. 1:**
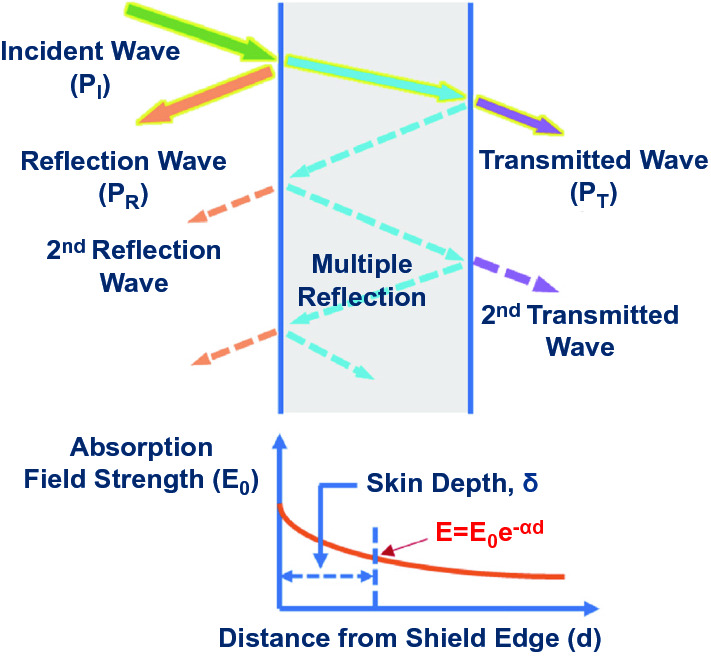
EMW propagation model in EMI shielding materials

The shielding efficiency (SE) can be used to evaluate the degree of suppression of EM energy for EMI shielding materials at a specific frequency [31]. Figure 1 shows the possible interaction between EMWs and shielding materials. When EMWs reach the surface of shielding material, it first interacts with the surface, then penetrates it and enters the inner part. A part of the EMWs are absorbed by the main body of material, resulting in absorption loss (*SE*_*A*_) [30, 32, 33]. Those EMWs that are not absorbed by the material are reflected by the material surface, resulting in reflection loss (*SE*_*R*_). When the absorbed EMWs travel to another interface of the shielding material, they are reflected again, followed by the energy dissipation in the shielding body, leading to multiple reflection loss (*SE*_*M*_). The three different types of losses, *i.e.*, *SE*_*R*_, *SE*_*A*_ and *SE*_*M*_, together make contributions to the attenuation of EMW [26], so the total SE of EMI (*SE*_*T*_) could be calculated from the sum of the above three effects, as shown in Eq. 1 [18, 23, 26, 34]:

1 $$\begin{array}{*{20}c} {{\text{SE}}_{T} = 10\log_{10} \frac{{P_{I} }}{{P_{T} }} = 20\log_{10} \frac{{E_{I} }}{{E_{T} }} = 20\log_{10} \frac{{H_{I} }}{{H_{T} }} = {\text{SE}}_{R} + {\text{SE}}_{A} + {\text{SE}}_{M} } \\ \end{array}$$

where ${\text{P}}_{{\text{I}}}$*,* ${\text{P}}_{{\text{T}}}$*,* ${\text{E}}_{{\text{I}}}$*,* ${\text{E}}_{{\text{T}}}$*,* ${\text{H}}_{{\text{I}}}$ and ${\text{H}}_{{\text{T}}}$ stand for the incident power, transmitted power, incident electric field intensity, transmitted electric field intensity, incident magnetic field intensity and transmitted magnetic field intensity, respectively. Usually, there are three situations that will occur, given the interactions between EMWs and shielding materials, as follows:

(i) When EMWs reach the material surface, the incident waves get reflected due to the discontinuous impedance on the surface of the material in contact with the air [26]. This reflection only requires the discontinuity of the impedance on the intersecting surface [31, 35]. After simplifying Fresnel’s equation for a highly conductive shielding materials, the reflection loss from the front to the back of the shield can be expressed as follows [36, 37]:

2 $$\begin{array}{*{20}c} {{\text{SE}}_{R} \left( {dB} \right) = 20\lg \frac{{\left( {\eta + \eta_{0} } \right)^{2} }}{{4\eta \eta_{0} }} = 39.5 + 10\lg \frac{\sigma }{2\pi f\mu } } \\ \end{array}$$

where *η*_*0*_ and *η* are the impedances of the free space and shielding material, respectively, *μ*, *σ* and *f* represent the permeability, conductivity of shielding material and incident EMW frequency, respectively. Obviously, *SE*_*R*_ increases with improving conductivity, indicating that the electrical conductivity is extremely important to achieve strong reflection loss for shielding material. However, the reflection loss is not only affected by conductivity. The EMW frequency and permeability of the shielding layer also play a part.

(ii) A part of the EMWs may not be reflected while entering the material. They would be gradually converted into the forms of dielectric loss, magnetic loss and conduction loss during the propagation process [26, 38–42]. This consumption is called absorption attenuation. Generally, the absorbing material can completely absorb the EMWs inside under normal circumstances. Magnetic permeability and dielectric constant are always used to quantitatively reflect the transmission and reflection properties of absorbing materials, respectively [43–46]. The *SE*_*A*_ for conducting and non-magnetic shielding materials could be expressed [37, 47]:

3 $$\begin{array}{*{20}c} {{\text{SE}}_{A} \left( {dB} \right) = 20\lg e^{\alpha d} = 20\left( {\frac{d}{\delta }} \right)\lg e = 8.68\left( {\frac{d}{\delta }} \right) = 8.7d\sqrt {\pi f\mu \sigma } } \\ \end{array}$$

where *α* is the attenuation constant and *δ* is the penetration or skin depth. This is a useful parameter for shielding and means the distance below the surface where the electric field intensity is reduced to 1 e^−1^ of the intensity of initial incident wave [48]. The conductivity and thickness are the main factors of absorption, while permeability and permittivity determine absorption loss [49].

(iii) When the EMWs that are not consumed inside the material reach another surface of the material, they again encounter the interface between the material and the air, and then return to the interiors of the material again [50, 51]. This kind of reflection is called multiple reflection [52–54]. Multiple reflections from the front and back of the shielding material lower EMI SE. *SE*_*M*_ could be calculated as follows [37]:

4 $$\begin{array}{*{20}c} {{\text{SE}}_{M} \left( {dB} \right) = 20\lg \left( {1 - e^{ - 2\alpha d} } \right) = 20\lg \left( {1 - e^{{ - \frac{2d}{\delta }}} } \right)} \\ \end{array}$$

*SE*_*M*_ is highly relied on thickness, and when thickness is near or greater than skin depth, or when *SE*_*T*_ reaches above 15 dB, *SE*_*M*_ can be ignored. However, when the skin depth thickness is larger than the thickness, multiple reflections must be considered when studying the shielding effectiveness [55–58]. The shielding capability can be enhanced by increasing additional interfaces within the shielding material. An interface with mismatched impedance characteristics results in additional internal scattering, also known as internal multiple reflection, which increases the absorption loss [59]. Internal scattering should be different from aforementioned multiple reflection The former caused by additional internal interfaces within the shield greatly increases absorption loss and overall shielding effectiveness, while multiple reflections occur between front and rear surfaces of the shield reduce shielding effectiveness [60–63].

As depicted in Fig. 1, the mechanisms of shielding EMWs in the medium through realizing the loss of EMWs are the reflection loss, multiple reflection loss and absorption loss [46, 47, 64]. For non-magnetic media, a continuous conductive path is formed on the surface that could result in an effective EMW reflection loss. The charged dipole and current path of the medium can effectively convert the energy of the EMWs into other energies (such as heat), realizing electromagnetic loss [65–69]. For magnetic media, the resonance or deflection of the magnetic dipoles mainly contributes to EMW absorption loss (*SE*_*A*_). The mechanisms of EMI shielding in the medium with multiple heterogeneous interfaces are mainly dominated by multiple reflection losses [64, 70–74]. Through the internal multiple reflections of EMWs that increase the propagation distance in the medium, the EMWs are attenuated [75–78].

At present, vector network analyzers are generally used for the EMI shielding ability test. Incident waves and transmitted waves can be represented mathematically by S-parameters ($S_{11}$ and $S_{21}$). Therefore, it is possible to calculate the ability of the material to reflect, absorb and shield EMWs according to Eqs. 5–10 [79]:

5 $$\begin{array}{*{20}c} {R = \left| {S_{11} } \right|^{2} } \\ \end{array}$$

6 $$\begin{array}{*{20}c} {T = \left| {S_{21} } \right|^{2} } \\ \end{array}$$

7 $$\begin{array}{*{20}c} {A = 1 - R - T} \\ \end{array}$$

8 $$\begin{array}{*{20}c} {{\text{SE}}_{R} = - 10\lg \left( {1 - R} \right)} \\ \end{array}$$

9 $$\begin{array}{*{20}c} {{\text{SE}}_{A} = - 10\lg \left( {\frac{T}{1 - R}} \right)} \\ \end{array}$$

10 $$\begin{array}{*{20}c} {{\text{SE}}_{T} = {\text{SE}}_{R} + {\text{SE}}_{A} = - 10\lg T} \\ \end{array}$$

where *R*, *A* and *T* represent the energy coefficients reflected, absorbed and transmitted, respectively, revealing the true EMW energy loss. However, *SE*_*R*_, *SE*_*A*_ and *SE*_*T*_ represent the ability to reflect, absorb and total shield EMWs, respectively. These two sets of indicators are easy to be misunderstood, because EMI shielding materials with high absorption capacity (*SE*_*A*_) do not necessarily absorb most of the energy of EMWs. This is because EMWs could only enter the material after reflection [80–83]. The reflection capacity (*SE*_*R*_) describes the ratio of the reflected energy to the incident energy, and the absorption capacity (*SE*_*A*_) describes the ratio of the absorbed energy to the energy entering the material. Obviously, the denominators are different when calculating *SE*_*R*_ and *SE*_*A*_.

### Index for "Green EMI Shielding"

For EMI shielding, a problem that cannot be ignored is that high-performance EMI shielding materials are usually composed of highly conductive materials, which can cause very strong secondary reflections. The strong reflected waves generate more significant electromagnetic radiation through mutual superposition and mutual interference, creating an additional adverse EM environment. This issue should be considered in EMI shielding materials seriously, but unfortunately it is usually neglected [84, 85].

With rapid development in human society, the emphasis should also be laid on green shielding of EMI, where "green" means less harm on external and/or internal environment of materials [84]. The ultimate goal of green electromagnetic interference shielding materials is to obtain low reflection and high shielding capability. This means that electromagnetic waves are mainly consumed in the form of absorption to protect the electronics on both sides of the shielding material. Cao et al. proposed the concept of green index (*g*_*s*_) for the first time and expounded the concept of "green shielding" materials [29]. Besides, they summarized the new requirements for "green EMI shielding" materials and gave the analytical method that defines the green index (*g*_*s*_) with the corresponding formula (Eq. 11):

11 $$\begin{array}{*{20}c} {{\text{g}}_{{\text{s}}} { = }\frac{{1}}{{{\text{S}}_{{{11}}}^{{2}} }} - \frac{{{\text{S}}_{{{21}}}^{{2}} }}{{{\text{S}}_{{{11}}}^{{2}} }} - 1} \\ \end{array}$$

where *S*_*11*_ and *S*_*21*_ represent input reflection coefficient and the transmission coefficient from input to output, respectively. S_11_ and S_21_ are expressed as follows (Eqs. 12 and 13):

12 $$\begin{array}{*{20}c} {{\text{S}}_{{{11}}} { = }\frac{{{\text{r}}\left( {{1} - {\text{e}}^{{ - {\text{i2nk}}_{{0}} {\text{d}}}} } \right)}}{{{1} - {\text{r}}^{{2}} {\text{e}}^{{ - {\text{i2nk}}_{{0}} {\text{d}}}} }}} \\ \end{array}$$

13 $$\begin{array}{*{20}c} {{\text{S}}_{{{21}}} { = }\frac{{{1} - {\text{r}}^{{2}} }}{{{1} - {\text{r}}^{{2}} {\text{e}}^{{{\text{ - i2nk}}_{{0}} {\text{d}}}} }}{\text{e}}^{{ - {\text{ink}}_{{0}} {\text{d}}}} } \\ \end{array}$$

where n, k_0_ and d represent refractive index, vacuum wave number and sample thickness, respectively. *R* could be obtained from Eq. 14:

14 $$\begin{array}{*{20}c} {{\text{r}}\;{ = }\;\frac{{{\text{Z}} - {1}}}{{\text{Z + 1}}}} \\ \end{array}$$

where Z is the impedance matching of sample, and could be calculated by Eq. 15:

15 $$\begin{array}{*{20}c} {Z = \sqrt {\frac{{\mu_{r} }}{{\varepsilon_{r} }}} tanh\left[ {\frac{2j\pi fd}{c}\sqrt {\mu_{r} \varepsilon_{r} } } \right] \approx \sqrt {\frac{{\mu_{r} }}{{\varepsilon_{r} }}} } \\ \end{array}$$

In addition, S_11_ and S_21_ are related to frequency (*f*), temperature (*T*) and sample thickness (*d*). Therefore, g_s_ can be described by Eq. 16:

16 $$\begin{array}{*{20}c} {g_{s} = \frac{1}{{\left| {S_{{11}} (f,{\text{ }}T,{\text{ }}d)} \right|^{2} }} - \frac{{\left| {S_{{21}} \left( {f,{\text{ }}T,{\text{ }}d} \right)} \right|^{2} }}{{\left| {S_{{11}} \left( {f,{\text{ }}T,{\text{ }}d} \right)} \right|^{2} }} - 1{\text{ }} = {\text{ }}g_{s} (f,{\text{ }}T,{\text{ }}d)} \\ \end{array}$$

The "green" materials depend on two key factors including the effective shielding effect (SE) and outstanding impedance matching and absorption loss.

High SE means that human being or working space (internal environment) can be protected from electromagnetic radiation, while good impedance matching could promote the absorption and restrict the secondary reflection of EMW, improving the *SE*_*A*_ for high *g*_*s*_ [85–87]. Generally, efficient shielding materials usually show a SE of ≥ 30 dB and a *g*_*s*_ of ≥ 1 [29]. The "green EMI shielding" materials should not only reduce the transmission of EMWs, but also dissipate EMWs as much as possible to achieve "green EMI shielding."

## Construction Strategies for Flexible EMI Shielding Materials

With the increasing use of highly integrated portable electronic devices, flexible EMI shielding devices are expected to be thin and light [88–90]. It is also believed that satisfactory electrical conductivity, complex permittivity and permeability are critical factors toward efficient EMI shielding. Meanwhile, materials with hierarchical structures (like porous, hollow-like) show great potential in EMI shielding. "Green EMI shielding" effects achieved in the materials themselves will cause less additional environmental hazard from the lower secondary reflection. Most of the EMWs radiated into the materials will be consumed by multiple reflections [76, 91].

**Fig. 2:**
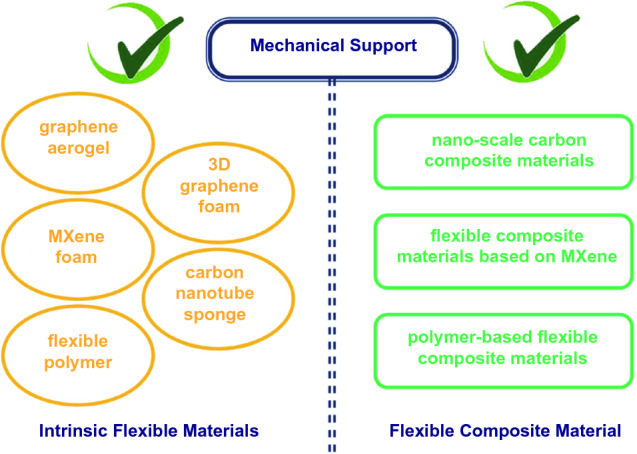
Classification of flexible EMI shielding materials

Some materials inherently possess good flexibility such as high degrees of bendability, twistability and foldability. Otherwise, they have to be combined with mechanical support materials to achieve flexible shielding. Therefore, depending on whether mechanical reinforcement is required, flexible shielding materials could be divided into two categories, including intrinsically flexible materials (graphene aerogel, 3D graphene foam, MXene foam, carbon nanotube sponge and fibrous polymer) and flexible composite material (nanoscale carbon composite flexible materials, flexible MXene-based composite materials and polymer-based flexible composite materials), as illustrated in Fig. 2 [92].

### Intrinsically Flexible EMI Shielding Matrixes

Intrinsically flexible EMI shielding matrixes are required to possess not only outstanding EMI shielding capabilities but also good flexibility. Aerogels, sponges, films or foams with highly porous 3D network composed of ultrahigh contents of gas phases and a solid matrix [93] show decent mechanical properties. Due to their unique physical characteristics including ultralow density, large openings as well as ultrahigh surface area, they can be promising candidates of intrinsically flexible EMI shielding materials.

#### Versatile Nanocarbon Matrixes from Aerogels, Sponges, Films to Foams Based on Graphene and CNTs

**Fig. 3:**
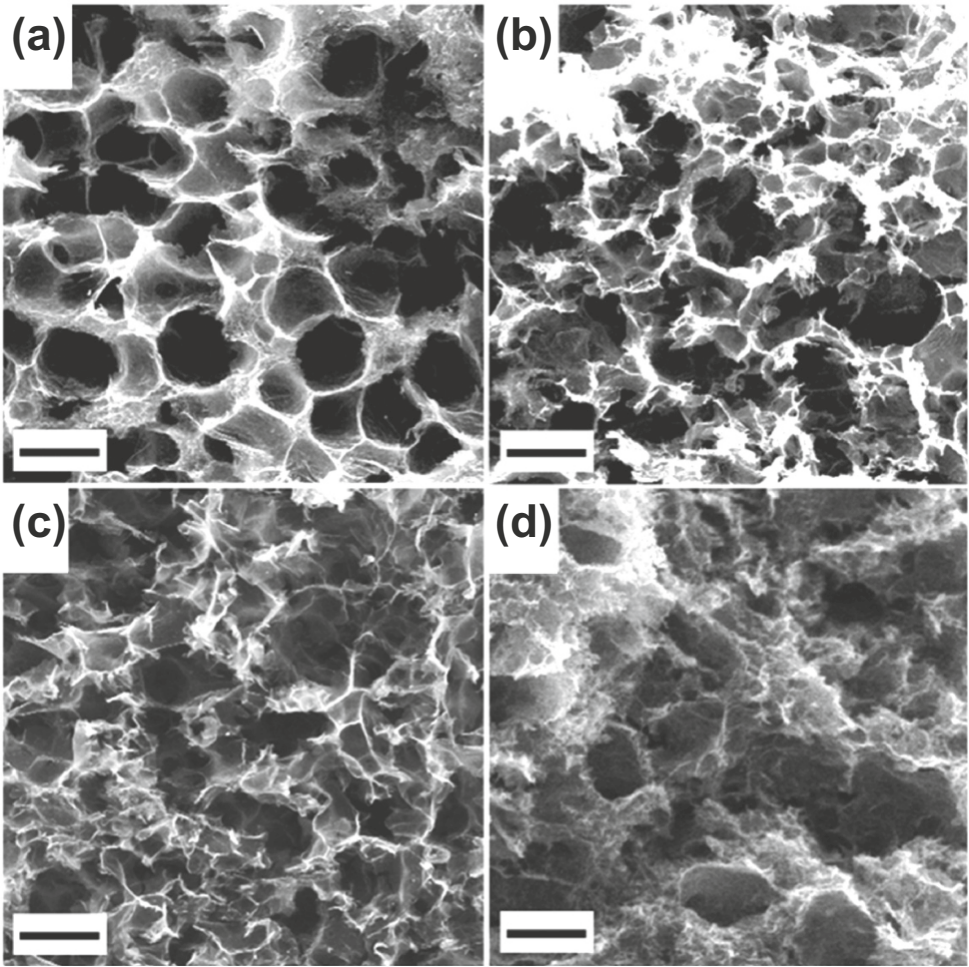
**a-d** SEM micrographs of graphene aerogels at different reduction temperatures: non-treated (**a**), 400 (**b**), 600 (**c**) and 1000 °C (**d**) under Ar/H_2_ 1:0.15 atm. The scale bar denotes 200 μm [97]; Copyright © 2019 Elsevier Ltd

Graphene aerogel has been utilized for flexible shielding material due to its lightweight, extremely high conductivity and mechanical stability, and unique 3D microporous structure [80, 94]. The large number of internal pores and free space in the graphene aerogel ensure low density and multiple reflections between the 3D carbon material and air [95]. In addition, the high conductivity of graphene materials increases the relative complex permittivity and *SE*_*R*_, thus enhancing the EMI shielding performance [96]. Marta et al*.* [97] prepared highly porous graphene aerogels via the improved hydrothermal treatment method. In a typical hydrothermal process, graphene oxide (GO) nanosheets are reduced and assembled around the hexane droplets. After two rounds of freeze-drying process, the hierarchical and porous structures with ~ 225 μm large pores and ∼5 μm small pores are obtained (Fig. 3a-d). The shielding SE of as-synthesized material is evaluated at 8–18 GHz. It is found that the transmittance is less than 5% of input EMW energy for all porous samples.

**Fig. 4:**
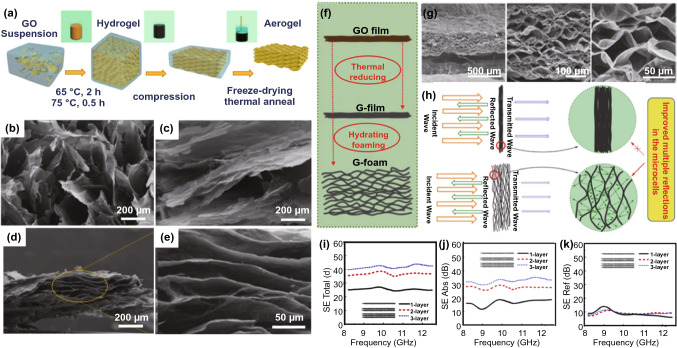
**a** Schematic illustration of the TGA fabrication procedure; SEM images of **b** TGA-C100%, **c** TGA-C40%, **d, e** TGA-C5% [126]; Copyright © 2020 American Chemical Society; **f** schematic representation of the fabrication process of G-film and G-foam; **g** SEM images showing microcellular structure in the cross section of G-foam; **h** schematic representation of multiple reflections in the microcellular structure of G-foam; **i-k** *SE*_*T*_, *SE*_*A*_ and *SE*_*T*_ of G-foam with different stacked layers in the frequency range of 8.2–12.5 GHz [100]. Copyright © 2016 Elsevier Ltd

The microstructure of graphene aerogels can also be tailored to improve electromagnetic loss [98]. Li et al*.* [99] prepared graphene aerogel by compressing graphene hydrogel mechanically, accompanied by freeze-drying and annealing (Fig. 4a). It was found that the microstructure of the hydrogel transformed from cellular to layered configuration after compressing (Fig. 4b-e), which plays a key role in dissipation of EMWs. The study also illustrated that the compressed graphene aerogel presented excellent conductivity of 181.8 S/m and EMI SE of 43.29 dB in X-band at the thickness of 2.5 mm, meaning ≥ 99.99% of EMWs have been shielded. To study the effects of different porous molds on EMI shielding, Shen et al*.* [100] systematically studied the EMI SE of graphene film (G-film) and microporous graphene foam (G-foam) (Fig. 4f-g). It is notable that changing the layered G-film into porous G-foam improves the performance of EMI shielding because of the *SE*_*M*_ by microporous structure at the matrix interface (Fig. 4h). Besides, rather than reflection, the thickness of the G-foam is a key factor in improving electromagnetic absorption (Fig. 4i-k). It is found that the SE of G-foam increased along with the increase in sample thickness, but such increment is not proportional. Crespo et al*.* [101] reported flexible carbon nanotube (CNT) sponge with < 0.02 g cm^–3^ density by CVD route for efficient shielding. Owing to its extremely lightweight, the specific SE (SSE) of it was found to be up to 1100 dB cm^3^ g^–1^, with a *SE*_*T*_ ≥ 20 dB throughout 1–18 GHz range, and capable of realizing shielding by absorption. Their remarkable net absorption ability favors "green EMI shielding" when incorporated into a multilayer structure to inhibit EMW reflection at the input interface. Thus, graphene aerogel, graphene film, microporous graphene foam and CNT sponge could be regarded as friendly "green EMI shielding" materials.

#### 2D MXene: Effective Assembly from Film to Foam

**Fig. 5:**
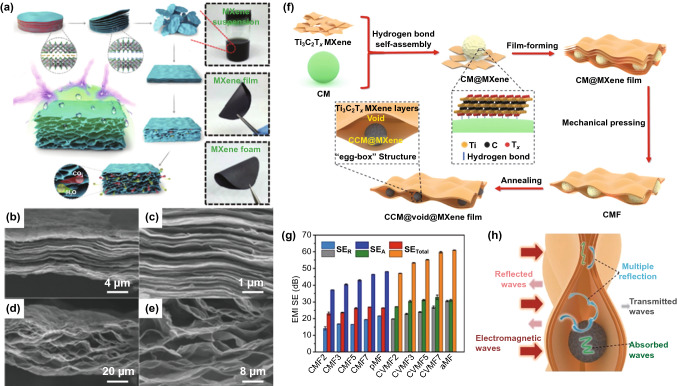
**a** Schematic illustration of the fabrication of the hydrophobic and flexible MXene foam. Cross-sectional SEM images of **b, c** the MXene film and **d, e** the MXene foam [105]. Copyright © 2017 WILEY‐VCH Verlag GmbH & Co. KGaA, Weinheim; **f** schematic illustration for the fabrication of CVMF; **g** comparison of average *SE*_*A*_, *SE*_*R*_ and *SE*_*T*_ for CMF, pMF, CVMF and aMF in X-band; **h** schematic description of the EMWs propagating across CVMF [106]. Copyright © 2020 Elsevier Ltd

As a promising type of two-dimensional (2D) materials, MXene perfectly interprets the superior electrical conductivity of the layered structure and shows reliable mechanical stability along with adjustable surface, which make it capable for a number of applications such as portable and wearable electronics, especially in the fast-growing field of flexible EMI shielding materials [102–104]. Liu et al*.* [105] first developed hydrazine-induced foaming technology to prepare hydrophobic MXene foam by assembly of MXene nanosheets into film and further into foam (Fig. 5a). It is found that when MXene film was converted to foam, its conductivity decreased as its thickness is increased (Fig. 5b-e). It exhibited higher shielding SE of 70 dB than 53 dB of pristine MXene film, due to its highly efficient EMW attenuation in its well-existed porous structure. Qian et al*.* [106] fabricated the unique egg-box-structured carbonized MXene films (Fig. 5f). It was shown that such structure with abundant interior voids could contribute to interfacial polarization and multiple reflection of incident EMWs, and finally promote the EMW absorption (Fig. 5g). Specifically, in Fig. 5h, EMW reflection of the MXene film was drastically increased because of the enhanced conductivity coming from the large amount of free electrons.

#### Non-Conductive Polymer Matrixes with Intrinsic Flexibility

Polymer-based materials with intrinsic flexibility have shown absorption-dominant EMI shielding capability, which is highly favorable in many application fields like military stealth [50]. In particular, conductive polymers possessing delocalized *p*-conjugated electrons display peculiar electronic properties, for example low ionizing potential and high electron affinity. The SE of conductive polymers roots in the moving charges as well as bound charges on the backbone [107]. Furthermore, conductive polymers possess easy preparation/processing, easy morphology/shape control, low density and tunable flexibility and conductivity. Nevertheless, for most of non-conducting polymers, they do not provide shielding effects. Therefore, the common approach is to introduce suitable conductive fillers to form polymer composites, which not only offer the possibility to adjust their physicochemical properties, but also afford the opportunity to modify the complex permeability and permittivity, and conductivity to optimize the shielding performance. Suitable filler or filler combinations are essential as well. These fillers would serve as the backbone to provide robust supports for ensuring the flexible structural integrity and effectively alleviating the structure destruction. Thus, the polymers without filler support are usually difficult for EMI shielding application directly. Oppositely, their composites could be more versatile with expanded application potential.

In brief, in order to achieve great flexibility and EMI shielding capacity, the conductivity and special network, usually in the form of aerogel, sponge, film or foam structure, are vital for intrinsic flexible substrate/matrix EMI shielding materials. It is highly feasible to achieve highly efficient EMI shielding materials from MXene, CNT, graphene, as well as many other carbon-based materials, which provide inspirations for advanced design of more flexible EMI shielding composites.

### Construction of Flexible Composite Architectures for Dielectric and Magnetic Synergy Effects

**Fig. 6:**
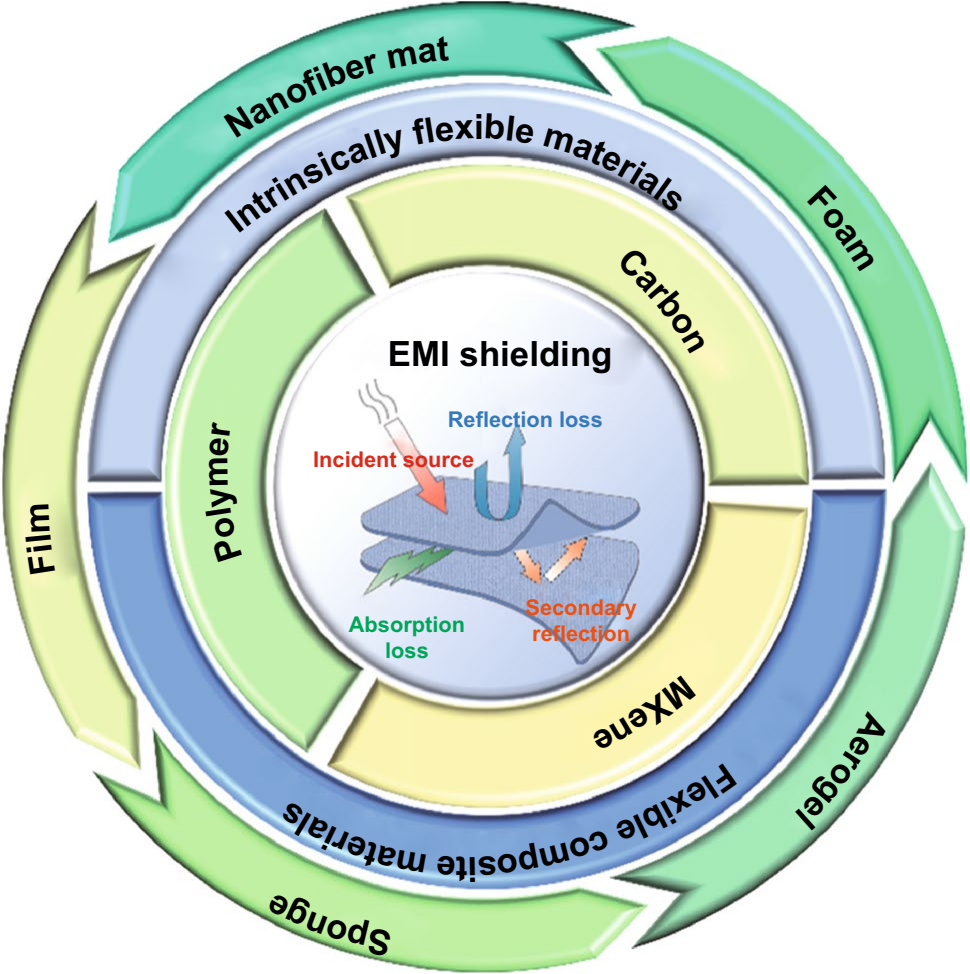
Application of flexible nanocomposite in EMI shielding

Nanocomplexing is regarded as a facile strategy to efficiently adjust conductivity and magnetic properties of the overall composites for superior EMI shielding performance. Also, it is popular strategy to construct flexible EMI shielding composites by combining intrinsically flexible bodies with mechanical supported fillers. Based on aforementioned flexible matrixes, reasonable dielectric or magnetic filling in these materials will greatly conduce to "green EMI shielding" by dielectric and magnetic synergy effects (Fig. 6).

#### Dilectric-type Complexing and Magnetic Filling for High *SE*_*A*_ Based on Flexible Nanocarbon Matrixes

As an alternative for conventional metal EMI shielding materials, carbon-based materials and their composite materials with high corrosion resistance, low density, suitable conductivity and easy processing feasibility have been intensively investigated. The nanocarbon-based materials including CNTs, graphene and other nanocarbons are widely applied to construct flexible EMI shielding composites [42, 108–113].

**Fig. 7:**
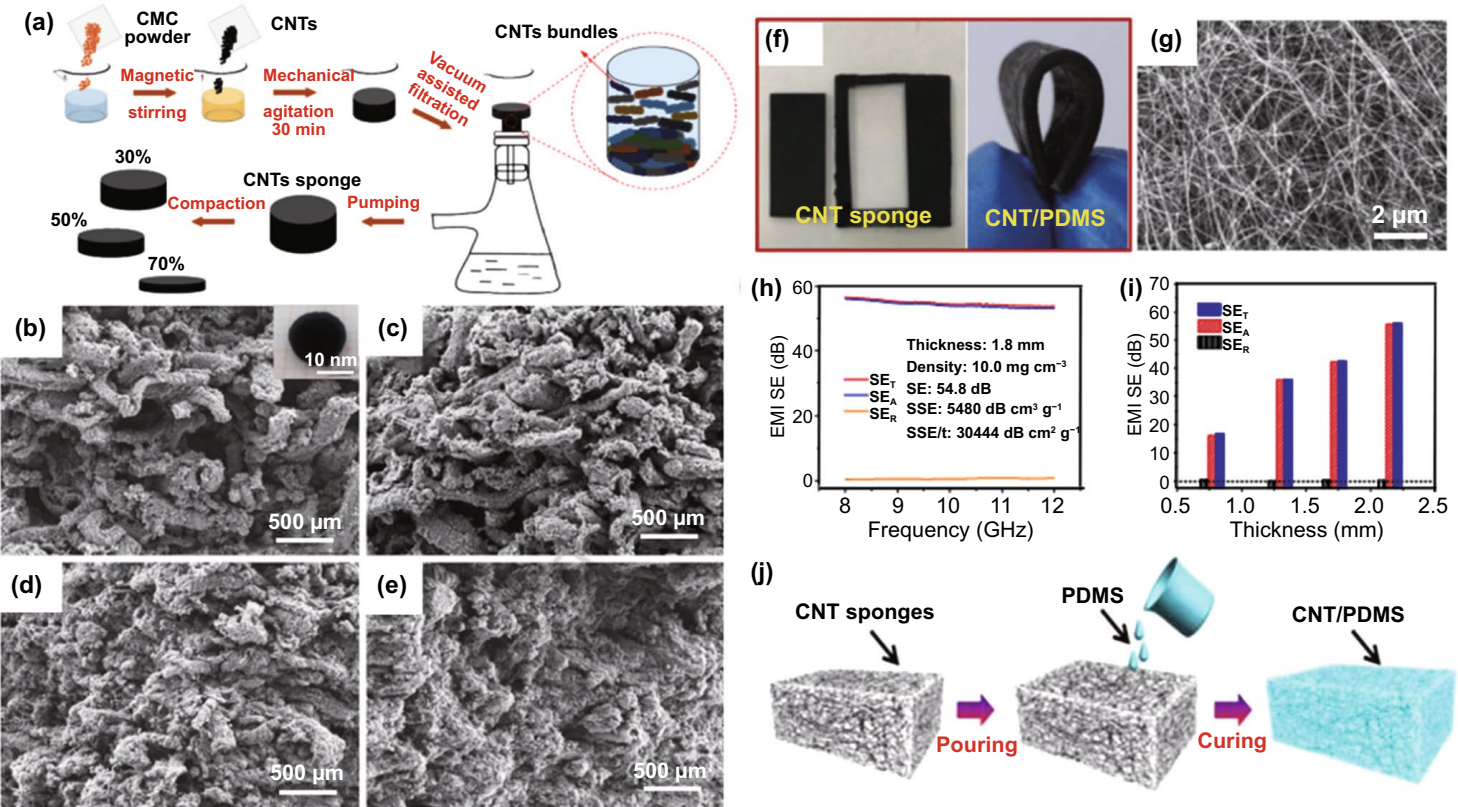
**a** Preparation process of composite CNT sponge with different compaction ratios. **b-e** SEM images of CNT sponges with varying compaction ratios [117]. Copyright © 2018 Elsevier Ltd. **f** Photograph of CNT sponges; **g** SEM image of the CNT sponge. **h** EMI SE of the CNT sponges in X-band. **i** Average *SE*_*T*_, *SE*_*R*_ and *SE*_*A*_ of CNT sponge with different thicknesses. **j** Schematic illustration of the fabrication of the CNT/PDMS nanocomposites [118]. Copyright © 2018 Elsevier Ltd

As typical one-dimensional (1D) material, CNT is regarded as a promising candidate for EMI shielding due to the high aspect ratio and conductivity. Moreover, the establishment of simple routes for constructing CNT-based 3D interconnected networks for mass production has attracted great interest [114–116]. Mei et al*.* [117] obtained CNT-based sponges with different compaction rates (0%, 30%, 50% and 70%) and studied their EMI SE in X-band (Fig. 7a). The microstructures of CNT sponges with different compaction ratios are displayed in Fig. 7b-e. With increased compaction degree, the pore size between the samples greatly decreases, showing a great impact on the sponge density, *i.e.,* the higher compaction degree is, the higher density of CNT sponge is, ranging from 11.1 to 24.4 vol%. It was also demonstrated that 70% compaction rate was beneficial to the buildup of denser and tighter CNTs networks as an interconnected conducting network and finally enhanced the SE of such CNT sponge/EP composite. Similarly, Lu et al*.* [118] directly used the flexible sponge-like CNTs composed of self-assembled, interconnected CNT skeletons as shielding films with 10.0 mg cm^−3^ density (Fig. 7f-g). The SE and SSE of the freestanding film with 1.8 mm thickness in X-band reach as high as 54.8 dB and 5480 dB cm^3^ g^−1^ (Fig. 7h-i). Further, these CNT sponges could also be combined with polymers for EMI shielding. Figure 7j presents the CNT/poly (dimethylsiloxane) (PDMS) nanocomposites as a typical case. Such CNT/PDMS film demonstrates satisfied SE of 46.3 dB at 2.0 mm of thickness with low CNT loading amount of < 1.0 wt%. After 1000 times of stretching or bending, the SE shows little change. These flexible, highly conductive and stable composites could be directly exploited for efficient EMI shielding coatings.

**Fig. 8:**
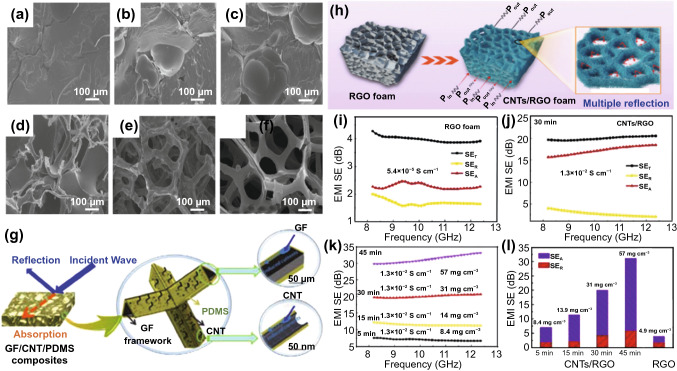
**a-e** SEM images of GF/PDMS composites with porosities of 9.3%; 28.9%; 51.5%; 73.2%; 90.8%. **f** SEM image of graphene layers after deposition on a Ni substrate. **g** Schematic illustration of effective EMI shielding by GF/CNT/PDMS hybrid composites through reflection and absorption [119]. Copyright © 2016 Elsevier Ltd. **h** Schematic illustration of the EMI shielding mechanism of CNTs/RGO foam composite. **i, j** *SE*_*T*_, *SE*_*A*_ and *SE*_*R*_ of the RGO and CNTs/RGO foam composites. **k** *SE*_*T*_, conductivity and density of CNTs/RGO foam composite with different reaction time. **l** The mean values of *SE*_*A*_ and *SE*_*R*_ of RGO and CNTs/RGO foam composite with different reaction time [116]. Copyright © 2019 Elsevier Ltd

The combination of CNTs and graphene foam can also achieve efficient shielding against EMWs while maintaining their flexibility. Sun et al*.* [119] combined PDMS, CNT and graphene foams (GF) with cellular structure to achieve great EMI shielding performance (Fig. 8a-f). Compared with GF/PDMS composite, the SE of GF/CNT/PDMS with the same porosity (90.8%) increases from 25 to 75 dB (Fig. 8g). However, it does not mean that the higher CNTs filling volume could result in the better EMI SE because there is no difference in the SE between GF/CNT/PDMS with 5 and 2 wt% CNT fillings. This is due to the synergistic effect of CNTs and GFs, which is manifested in the introduction of CNTs that greatly improves the EMW absorption capacity of the composites. On the one hand, the conductive network enabled by the GFs could provide the pathway for electromagnetic field-induced currents, while on the other hand, CNTs could add more interfaces for surface current attenuation. When the content of CNTs was further increased, the conductivity of the GF/CNT/PDMS composite could no longer be improved significantly due to the agglomeration effect. Therefore, the EMI shielding ability did not increase further. Similarly, Kong et al*.* [116] prepared porous CNTs/rGO foam composites for efficient EMI shielding (Fig. 8h). The introduction of CNTs increased *SE*_*R*_ and *SE*_*A*_ of the foam simultaneously and thus realized the enhanced EMW attenuation (Fig. 8i- j). The EMI SE of CNTs/rGO reached 31.2 dB at 2 mm thickness, and the SSE even reached 547 dB cm^3^ g^−1^ with an ultralow density of 57 mg cm^−3^ (Fig. 8k-l). Sundararaj et al*.* [41] combined CNTs, carbon nanofibers (CNFs) and carbon black (CB) nanoparticles (NPs) with acrylonitrile–butadiene–styrene (ABS) polymer, and found that the CNT/ABS nanocomposites showed the best EMI shielding performance on account of their higher aspect ratio and electrical conductivity.

**Fig. 9:**
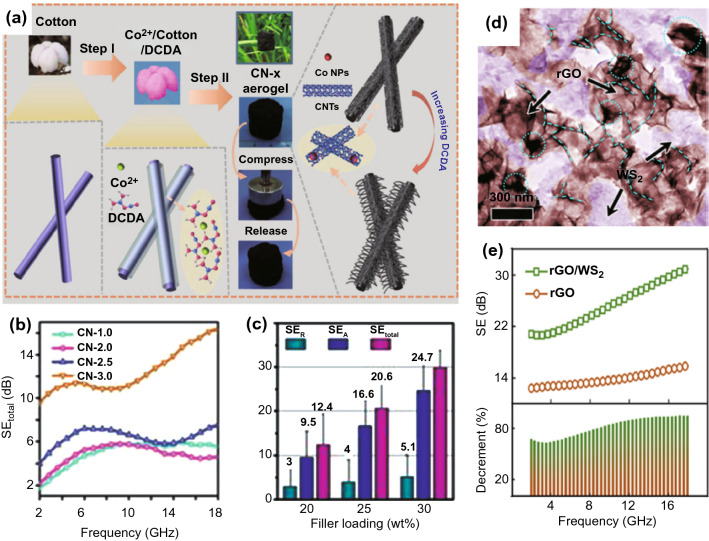
**a** Schematic graph of the formation process of CN-x composites. **b** The *SE*_*T*_ of all composites. **c** Shielding effective of *SE*_*R*_, *SE*_*A*_ and *SE*_*T*_ values of CN-3.0 composite with different filler loadings of 20, 25 and 30 wt% [74]. Copyright © 2019 WILEY‐VCH Verlag GmbH & Co. KGaA, Weinheim. **d** TEM images of WS_2_ − rGO architecture. **e** SE of WS2 − rGO at and comparison with rGO [37]. Copyright © 2019 ACS Publications

In spite of combination with each other among carbon materials to form composites, intrinsic carbon composition is usually limited by extremely high permittivity, resulting in the impedance mismatch. To promote the electromagnetic coupling of carbon materials and thus balance their impedance matching, magnetic particles or low permittivity of nanomaterials are exploited to be embedded or hybridized in the carbon materials. Cheng et al*.* [74] designed a lightweight and flexible composite aerogel, which was comprising of Co NPs anchored CNTs grown on cotton-derived micron-scale carbon fibers via a facile CVD method (Fig. 9a). It was revealed that the conductivity change of the sample could be controlled by adjusting the morphology of CNTs, *i.e.,* longer and straight CNTs contributed to improved conductivity, resulting in considerable SE of 29.8 dB (Fig. 9b). In contrast, short and curled CNTs with optimized conductivity could facilitate the *SE*_*A*_, resulting in a wide EAB of 5.08 GHz at 1.6 mm. Overall, when CNTs were filled at the amount of 25% and 30 wt% in matrix, the *SE*_*T*_ reached 20.6 and 29.8 dB, respectively, indicating their capability for shielding ≥ 99% incident EMWs (Fig. 9c). For obtaining "green EMI shielding," Zhang et al. [37] developed a novel dielectric-type WS_2_-rGO self-assembly architecture (Fig. 9d). The unique WS_2_-rGO gable structure also exhibited efficient and "green EMI shielding" within 2–18 GHz, with the SE > 20 dB, and the maximum shielding value of 32 dB (Fig. 9e). Endearingly, the green index (g_s_) was evaluated to be near 1.0. It is revealed that their multilayer structure and inherent dielectric properties, including synergistic relaxation and conduction, and multiple scattering within abundant voids together contributed to the efficient and green EMI SE.

**Fig. 10:**
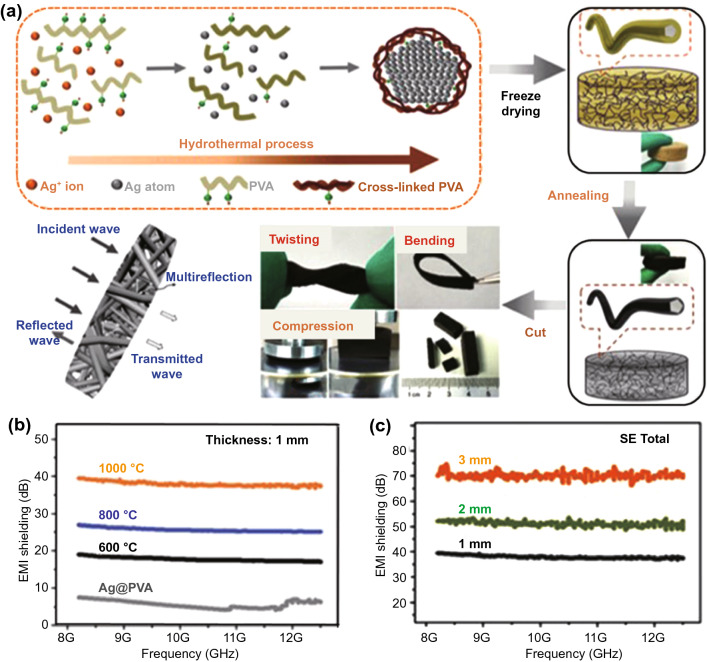
**a** Schematic illustration of fabrication of silver wire wrapped carbon core–shell (Ag@C) hybrid sponge and its application in EMI shielding. **b, c** EMI shielding performance of Ag@C-1000 sponge with different thicknesses and *SE*_*T*_ [120]. Copyright © 2018 WILEY‐VCH Verlag GmbH & Co. KGaA, Weinheim

Furthermore, Wan et al*.* [120] manufactured a flexible, lightweight and corrosion-resistant Ag nanowire-wrapped carbon (Ag@C) sponge (Fig. 10a), which exhibited ultrahigh EMI SE with superior mechanical stability and ultracompressibility. The Ag@C sponge with a low density of 0.00382 g cm^−3^ achieved 363.1 S m^−1^ conductivity and an ultrahigh SE of 70.1 dB within 8.2–18 GHz. Besides, the SE of the Ag@C sponge was positively correlated with the thickness and annealing temperature, suggesting that the conductive nanocarbon played a key role in EMI shielding (Fig. 10b-c).

Nanoscale carbon materials are good EMW dissipation materials, regarding their great electrical conductivity. At the same time, their easy processability ensures that they can be combined with other mechanical support materials easily or can be produced into a variety of microstructures. Particularly, porous materials can trap EMWs within their pores, promoting multiple reflections to achieve improved *SE*_*A*_. For flexible composite materials, the high-aspect-ratio conductive fillers and segregated structure are beneficial to the low percolation threshold; thus, high SE is achievable at relatively low filler loading levels than the randomly distributed systems. Moreover, the incorporation of dielectric materials can further increase the *SE*_*T*_ due to dielectric loss. Similarly, incorporation of magnetic materials can also increase the *SE*_*T*_ owing to interfacial polarization, eddy current loss and magnetic losses involving the magnetic domain movement, relaxation of the magnetization, etc.

#### Polymer Insertion and Ion Doped in Highly Conductive MXene

MXene is a distinguished 2D transition metal carbide and/or nitride (M_n+1_X_n_T_x_) where M is an early transition metal and X stands for C or N [22]. The superior electrical conductivity and mechanical properties related to metal ions, and the facile insertion of organic molecules and ions, together make MXene good candidates for EMI shielding [121]. As a highly conductive filler, it has attracted tremendous attention in producing EMI shielding composites. In combination with polymers fiber or other fibers, MXene composites can achieve outstanding mechanical properties. MXene Ti_3_C_2_Tx is often incorporated into different polymer matrices to improve its tensile strength, while maintaining good electrical conductivity under low polymer loadings.

**Fig. 11:**
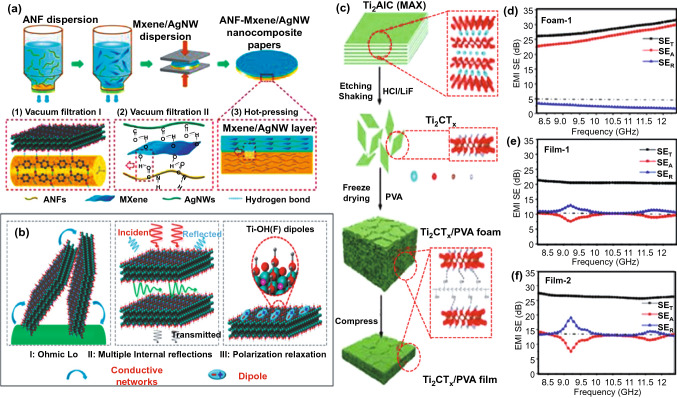
**a** Schematic diagram for the fabrication of double-layered ANF-MXene/AgNW nanocomposite papers. **b** EMI shielding mechanism of the double-layered nanocomposite papers [99]. Copyright © 2020 American Chemical Society. **c** Illustration of the preparation process of Ti_2_CT_x_/PVA composite foam and film. **d** Proposed EMI shielding mechanism of MXene/PVA composites with a foam or film structure. **e, f** EMI shielding performances of Ti_2_CT_x_/PVA foam-1, film-1 and film-2 [60]. Copyright © 2019 American Chemical Society

The EMI SE of freestanding MXene-based paper was studied by Ma et al*.* [99]. They prepared aramid nanofibers-Ti_3_C_2_T_x_/Ag nanowire (AgNW) composite paper with a double-layer structure, super-flexibility and high mechanical strength, and found that higher content of MXene/AgNW led to stronger SE. The MXene/AgNW double-layer nanocomposite paper exhibited high conductivity as well as excellent electrical stability and even maintained excellent shielding performance (~ 80.0 dB, 91 mm, X-band) after repeated bending and stretching (Fig. 11a-b). Jiang et al*.* _ENREF_22 [122] explored an efficient EMI shielding composite paper based on cellulose /MXene (Ti_3_C_2_T_x_) via a simple dip coating approach. It is found that with the increase in the number of coatings, the conductivity of the composite paper increases remarkably from 0 to 2756 S m^−1^ attributed to the gradual formation of Ti_3_C_2_T_x_ conductive networks. After seven times of dip coating, the SE of the composite reaches 43 dB, which was much better than that of the composite paper without dip coating. Even after 2000 cycles, the EMI SE could still attain exceeding 90% (42.1 dB), indicating its broad application for next-generation flexible devices. Although MXene shows great potential for construction of conductive papers, it is still challenging to achieve satisfactory EMI SE with lowered amount of MXene. Feng et al*.* [123] systematically studied the influence of Mn^2+^ insertion on composite films and found that the introduction of Mn^2+^ significantly enhanced the shielding performance. Due to the ion bridging effect between MXene nanosheets, the overall electrical conductivity could be ultrahigh (4268 S m^−1^) with less amount MXene by Mn ions, which is nearly three times than original Ti_3_C_2_T_x_ films (1894 S m^−1^). The SE of Mn ion modified film could reach as high as 69 dB at 9.4 GHz.

As set forth, foaming is considered as an efficient technique to build up the shielding materials with high flexibility and lightweight. Xu et al*.* [60] produced porous Ti_2_CT_x_ MXene/polyvinyl alcohol (PVA) composite foam (Fig. 11c). The calculated SSE reached 5136 dB cm^2^ g^−1^ with ultralow PVA filling of 0.15 vol% and ultralow *SE*_*R*_ of < 2 dB. After the foam was compressed into a thin film, the EMI shielding mechanism was changed from *SE*_*A*_-dominated to *SE*_*R*_-dominated mechanisms (Fig. 11d-f). Comparative experiments confirmed that internal multiple reflection, porous structure and dipole polarization show synergistic effects on improving *SE*_*A*_ toward excellent EMI SE. However, the introduced bubbles also easily destroyed the conductive networks in the composite, which leaded to the degradation of EMI SE. Zhao et al*.* prepared Ti_3_C_2_T_X_-MXene/rGO aerogel with successive core–shell structures via hydrothermal assembly [124]. The porous structure and superior conductivity (1085 S m^−1^) together enhanced the Ti_3_C_2_T_x_ composite aerogel an outstanding SE of 50 dB across X-band, which was a milestone record for the composites with similar MXene loading amount. Meanwhile, the composite aerogel also possessed stable EMI SE for EMWs from different directions attributed to the highly ordered lattice structure. Such assembly of three-dimensional (3D) porous structure can greatly promote the practical utilization of MXene-based composites for EMI shielding devices.

Although the EMI shielding performance is prominent for bare MXenes, their dielectric constants and conductivity are too high; thus, the resulted *SE*_*R*_ is too high while *SE*_*A*_ is low, which consequently cause undesired secondary pollution of EMWs. Incorporation of multiple components in the MXene-based composites could reduce the overall conductivity and EM reflection, resulting in moderate permittivity and increased dielectric loss. When the complex permeability and permittivity are close to each other, they could exhibit the best EMI *SE*_*R*_ and *SE*_*A*_ at the same time. In summary, combination of MXene with mechanical support materials such as polymers and carbon materials can take full advantages of the electromagnetic dissipation capability of MXene while achieving flexibility.

#### Reinforced Flexible Polymer Composites by Mechanical Supported Matrixes

Polymers are widely used for EMI shielding due to the satisfactory flexibility, corrosion resistance, lightweight and cheap price, while most polymers have poor mechanical property and inherently low electric conductivity, which limit their practical applications. Therefore, it is necessary to mix polymers with conductive and high-strength fillers to obtain desired EMI SE [125–128]. For non-conducting polymer, filling is usually employed to retain reinforced bulk polymer composite, which refers to the composite material consisting of polymer matrix and particle/fiber-type conductive fillers, and the combination of the two into it. Das et al*.* [129] reported a bulk polymer composite with short carbon fiber (SCF) and carbon black (CB) as filler, and vinyl acetate (EVA) as matrix. Correspondingly, the EMI shielding performance of SCF/EVA, CB/EVA and SCF/CB/EVA composite were studied. As a result, it is found that composites with SCF filler exhibited more superior EMI shielding performance than the composites with CB filler. The reason was also confirmed as SCF showed better dispersion in matrix, thus enhancing the SE. Notably, the shielding effect is inconsistent at Ku band (8–12 GHz) as the frequency increases. Oppositely, it increases slowly as the frequency increases in the lower-frequency range (100–2000 MHz). These carbon fillers could effectively promote the delocalization of the charge carriers and enhance the structural order of the polymer chains, thus facilitating the conductivity.

**Fig. 12:**
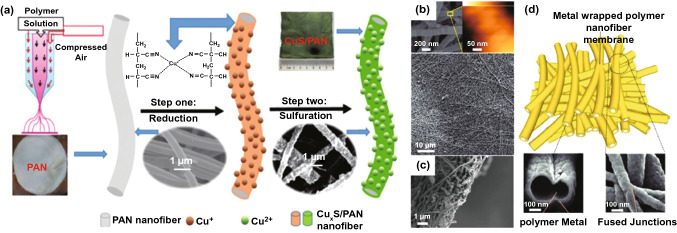
**a** Schematic diagram for the preparation of Cu_x_S/PAN nanofiber mat [131]. Copyright © 2019 WILEY‐VCH Verlag GmbH & Co. KGaA, Weinheim; **b-d** SEM and AFM (the scale bar for height is 0–240 nm) images of the Cu-wrapped nanofibers in the cellular membranes; cross-sectional SEM image of the membranes; Schematic and microstructure of the cellular membranes composed of high-conjunction Cu-wrapped polymer (PDA precoated nylon) nanofibers [132]. Copyright © 2020 WILEY‐VCH Verlag GmbH & Co. KGaA, Weinheim

The composite of metal and non-conductive polymer is a feasible strategy for flexible EMI shielding [130]. Li et al*.* [131] prepared flexible Cu_x_S/polyacrylonitrile (PAN) nanofiber mats (Fig. 12a). Such Cu_x_S/PAN mats demonstrated excellent EMI shielding capability (29–31 dB) at low frequencies (500 -3000 MHz). Besides, Zeng et al*.* [132] designed membranes based on easy polydopamine (PDA)-assisted Cu or Ag deposition on electro-spun PDA polymer nanofibers. The PDA layer served as a substrate allowing the growth of ordered Cu NPs to form continuous layers, with the root mean square surface roughness of ≤ 9.2 nm, suggesting distinct core–shell structure in the membrane (Fig. 12b-d), which enabled the high conductivity and EMI SE of as-obtained membranes. Therefore, by effectively utilizing the interaction between cellular structure of metal and polymer nanofibers, excellent flexibility and conductivity as well as ultrahigh EMI SE could be achieved. The SE of membrane (2.5 µm of thickness, 1.6 g cm^−3^ of density) was up to 53 dB with a broad frequency range. The SE of 44.7 dB was achieved at the lowest thickness (1.2 µm) with normalized SSE as high as 232,860 dB cm^2^ g^−1^. In addition, Shen et al*.* have also exploited PDA functionalization to construct polymer films based on Ag@CNTs hybrids [133]. A flexible and highly conductive Ag@PDA@ carbon nanotube–polyvinyl alcohol (PVA) film was formed with the addition of well-dispersed carbon nanotubes and additional silver particles. Compared with the pure carbon nanotubes (21 dB), the shielding efficiency was significantly increased to 42.75 dB for the composite film. PDA polymerization time controls the size of silver particles, the formation of effective conductive network and conductive/interfacial polarization-induced loss mechanisms determine the shielding performance of the film.

**Fig. 13:**
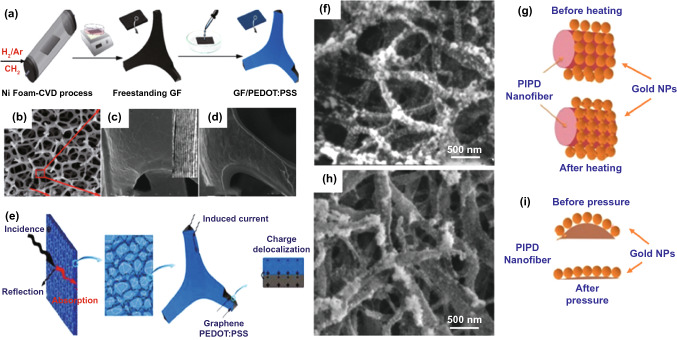
**a** Schematic procedure of the preparation of GF/PEDOT:PSS composites. SEM and TEM images of GFs **b, c** before and **d** after PEDOT:PSS coating. **e** Schematic illustration of EMI shielding mechanisms [134]. Copyright © 2017 American Chemical Society. **f-i** SEM images of the PIPD-g-PDDA/Au6 composites; scheme of the gold network changes during the heating process; SEM images of the PIPD-g-PDDA/Au6 composites pressed at 3 MPa for 2 h; Scheme of the gold network changes during the pressing process [136]. Copyright © 2017 Royal Society of Chemistry

In contrast, conductive polymers like polyaniline (PANI), polyfuran (PF), polythiophene (PTH), polypyrrole (PPy), poly(3,4-ethylenedioxythiophene) (PEDOT), polyparaphenylene (PPP), polyacetylene (PA) and poly(p-phenylene vinylene) (PPV), not only maintain decent mechanical properties, but also show outstanding electrical conductivity, making them suitable candidates as flexible EMI shielding agents. Combined with other electrically conductive materials, it is expected that EMWs can be dissipated effectively due to the synergistic effect. Wu et al*.* [134] reported ultralight EMI shielding composites utilizing the GF/poly(3,4-ethylenedioxythiophene):poly(styrenesulfonate) (PEDOT:PSS) (Fig. 13a). An ultralight porous composite structure with ultralow density of 0.0182 g/cm^3^ was gained when GF mass fraction is 58% (Fig. 13b-d). Owning to the good conductivity and porous structure, the composites delivered excellent EMI shielding performance (91.9 dB in SE, 3124 dB cm^3^ g^−1^ in SSE). And as shown in Fig. 13e, the charge delocalization in highly conductive networks plays a key role by generating the local eddies under an alternating EM field.

Although conductive carbon-based foams show lightweight and high EMI SE [135], their flexibility and conductivity seem insufficient as compared to those of metal. Alternatively, the combination of flexible skeleton of polymer and highly conductive networking species is expected to achieve high EMI shielding performance. Li et al*.* [136] constructed a porous polymer nanofiber material with a density of only 0.26 g cm^−3^ via assembly of Au NPs on a poly(pyridobisimidazole) grafted polydimethyl diallyl ammonium (PIPD-g-PDDA) backbone. It is revealed that the lower the Au content applied, the faster the conductivity declined. For high-Au-content PIPD-g-PDDA/Au film, the weight of the film decreases by about 0.8% after heating, with its surface morphology collapsed, as shown in Fig. 13f. The Au NPs were found to fuse together to form a solid gold network. Besides thermal treatment, compressing provides another possibility to promote the electrical conductivity of thin films. The electrical conductivity value of PIPD-g-PDDA/Au films rose to 22,240 ± 998 S cm^−1^ at 3 MPa pressure. The compressed morphology of the composite material was shown in Fig. 13h. Under compression, as the cross-linked structure of PIPD-g-PDDA NFs changed from round to flat, the Au NPs were arranged more closely, which reduced the contact resistance of Au network (Fig. 13i). It was also found that the shielding effect exceeds 64.9 dB in the nanocomposite material with only 20 mm thick and in the band of 250 MHz-1.5 GHz. By comparing and analyzing *SE*_*R*_, *SE*_*A*_ and *SE*_*M*_, the related shielding mechanism is revealed, suggesting that the PIPD-g-PDDA/Au composite materials were both absorptive and reflective to EMW within a certain frequency band, where the *SE*_*R*_ was dominant for overall *SE*_*T*_. As discussed above, conductive dielectrics-based fillers show satisfactory permittivity and conductivity, low density, as well as suitable physicochemical properties, contributing not only to reliable physical/mechanical properties, but also to high EMI SE of the corresponding composites. The shielding mechanism depends on balanced combination of *SE*_*A*_ and *SE*_*R*_, rather than the *SE*_*R*_-dominant mechanism of metals.

As a comparison, we summarize EMI shielding properties of representative flexible EMI shielding materials from intrinsically flexible substrate to composite flexible matrix (Table 1). It seems that the foam structure could be a promising choice for highly efficient shielding materials. Furthermore, it is also an effective approach to design the shielding materials with multiphase interfaces, which could extremely improve the multireflections and promote EMWs absorption. Nonetheless, an in-depth exploration on the materials that possess both ultralow thicknesses, low density and high SE as well as large SSE, and on the mechanisms of shielding in different bands or even broadband is still highly required.

**Table 1:** Comparison of EMI SE of representative flexible EMI shielding materials

Materials	Thickness (mm)	Density (g cm^−3^)	EMI SE (dB)	EMI SSE (dB cm^2^ g^−1^)	Refs
PI	2.5	0.076	26.1–28.8	1373–1518	[80]
Graphene aerogel	2.5	–	43.29	–	[165]
Graphene aerogel	5	0.006	–	6743	[97]
Graphene foam	0.3	0.06	25.2	–	[100]
MXene foams	–	–	70	–	[105]
Microsphere@void@ MXene	0.01	–	46.51–59.76	18,637.14	[106]
CNTs/RGO foam	2	5.7	31.2	547	[117]
CNT sponge/epoxy	3	–	53.14	–	[118]
PIPD-g-PDDA/Au	0.02	–	66.9	15,890	[136]
GF/CNT/PDMS	–	–	75	833	[119]
CNTs aerogel	1.6	–	29.8	–	[74]
CNTs/RGO	2	–	31.2	547	[116]
Ag@C	3	0.00382	70.1	61,169	[120]
d-Ti_3_C_2_T_x_/r-CNFs	0.015	–	42.7	–	[22]
MXene/AgNW	–	–	80	3725.6	[99]
rGO/epoxy	–	0.06	38	500	[125]
PVDF/MWCNT/GNPs/Ni	0.3	–	43.7	–	[128]
GF/PEDOT:PSS	–	0.0182	91.9	3124	[134]
Cu-wrapped polymer nanofiber	0.0025	1.6	53	232,860	[132]
PAN/CNT/Fe_3_O_4_	1.5	–	59.85	–	[147]
PPy/MXene	1.3	–	90	1000	[140]
Cu_x_S/PAN	0.423	0.044	29–31	16,655.92	[131]
MCP-SiC composite paper	0.3	–	67	1–	[137]
AgNWs/cellulose films	0.0445	–	101	5571	[138]
PP/PDA/AgNPs/PDMS	–	0.263	71.2	270.7	[139]
AgNF	0.1	–	76	–	[143]
PEBAX/graphene	–	–	30.7	–	[145]
Fe_3_O_4_@Ti_3_C_2_T_x_/GF/PDMS	–	–	77–80	–	[146]

## Multifuctionality

Multifuctionality is inevitably a main direction for future development of EMI shielding materials. Particularly, the development of low-dimensional, nanoscale and functional EMI shielding agents could bring about advanced properties of materials. New functionalities related to EM properties in miniaturized EM devices occupy a lot of potential in the future development of various scientific and technological fields. Besides EMW absorbing and shielding, the new functionalities also include EMW filtering, sensing, optics and energy conversion and storage devices.

### Thermal Conductivity and Hydrophobicity

**Fig. 14:**
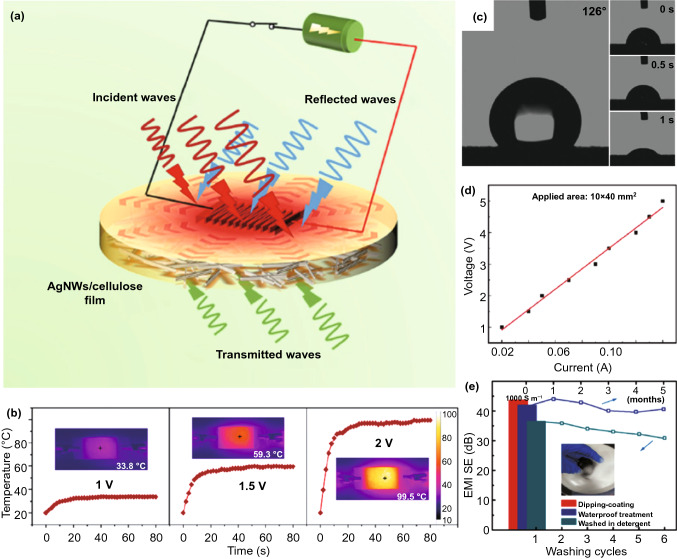
**a** Schematic diagram of multifunctional flexible AgNWs/cellulose films; **b** Joule heating performances [138]. Copyright © 2020 American Chemical Society. **c** Water-contacting angle measurements of M-textiles without (right) and with the coated silicone (left); **d** I–V curve of silicone-coated M-textile; **e** Effects of water-resistant treatment on the stability of EMI shielding performance [140]. Copyright © 2018 WILEY‐VCH Verlag GmbH & Co. KGaA, Weinheim

For modern flexible electronic devices, the effective heat dissipation during operation is essential, which ensures the reliability and service life of devices. Therefore, it opens an important direction for developing next-generation EMI shielding material with efficient heat conduction. Usually, the parameter thermal conductivity (TC) represents the ability of material to conduct heat, with its unit of W m^−1^ K^−1^, which means the capacity of material to transport a specific quantity of heat energy in 1 s via a plate of a specific area (1 m^2^) and thickness of (1 m) when its opposite face differs in temperature by 1 K. The development of EMI shielding materials should integrate the functionalities of high TC and high SE values. Chaudhary et al*.* [137] prepared multicomponent framework derived SiC composite paper with both strong EMI shielding effect (−67 dB at 10.3 GHz) and good TC of 6.5 W m^−1^ K^−1^, which were positively correlated with the amount of SiC. Similarly, Gu et al*.* [138] obtained AgNWs/cellulose films with outstanding mechanical strength and superior in-plane TC of 10.55 W m^−1^ K^−1^. Meanwhile, the SE was ultimately retained as ∼95 dB after bending for 1500 cycles. To further apply the AgNWs/cellulose films to thermal management, polyimide electrothermal films were pasted on AgNWs/cellulose films, and the variation in surface temperature was recorded (Fig. 14a). Unlike the sharp temperature increase when pure cellulose or air was used as heat sink, the maximum operating temperature fell to around 142 °C when 50 wt% AgNWs/cellulose film was utilized as heat sink, demonstrating its efficient heat dissipation during device operation. Additionally, the 50 wt% AgNWs/cellulose film also showed rapid response to Joule heating (Fig. 14b). When the input voltage is 1 V, the temperature did not increase greatly. However, the 1.5 and 2 V of input voltage caused rapid temperature increase of the AgNWs/cellulose film, which finally stabilized at 59.3 and 99.5 ℃, respectively.

Due to the requirement of water resistance for electronic devices, hydrophobicity becomes one of the emerging functions for EMI shielding materials. Gao et al*.* [139] prepared conductive polymer fabric composites (CPFCs) with high SE of 71.2 dB and an extremely high water-contacting angle (152.3°). More importantly, the super-hydrophobicity and EMI SE retained great stability after the abrasion/winding cycling tests. Zhang et al*.* [140] further designed and prepared a new fabric material, *i.e.,* silicone-coated PPy-modified MXene sheets embedded on poly(ethylene terephthalate) (PET) textiles. Besides excellent joule heating and EMI SE performance, the thin silicone coating offered high hydrophilic property (Fig. 14c). The silicon-coated textiles occupied a contact angle up to ≈126°, which to a large degree protected MXene from water-induced oxidation and afforded valuable waterproofing performance. The experimental results also showed that the flexible and multifunctional textiles had low resistance from nearly linear *I*–*V* curve, which ensure voltage-driven heating favoring the safety of operators (Fig. 14d). The EMI shielding efficiency of resultant multifunctional textile at a thickness of 1.3 mm was up to 90 dB. What is more, it could maintain great EMI shielding ability even after washing (Fig. 14e). Therefore, such versatile textile holds great potential in smart clothing allowing both EMI shielding and personal heat management.

### Transparency, Sensing and Multiple Functions

**Fig. 15:**
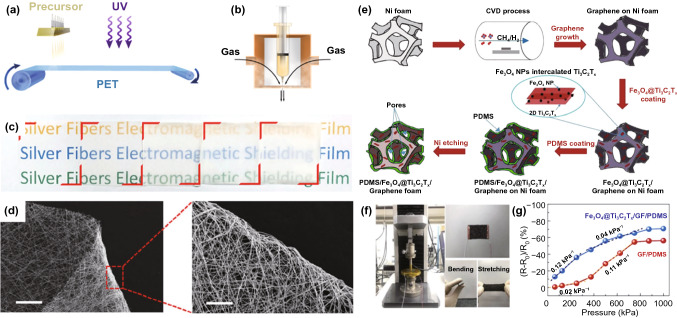
**a** Schematic diagram of the fabrication of AgNF by continuous blow spinning and in situ UV irradiation. **b** Partially enlarged detail of needle module shows the principle of blow spinning. **c** A photograph of as-prepared fiber film after different spinning time (from left: 1, 2, 5, 10 and 20 min). **d** SEM image of a bended AgNF film and its partially enlarged areas, With scale bars of 300 and 50 μm, respectively [143]. Copyright © 2019, Springer Nature. **e** Schematic diagram of the fabrication procedure for Fe_3_O_4_@Ti_3_C_2_TX/GF/PDMS composite. **f** Images of the equipment used for pressure sensing measurement (left), GF-based composite with two electrode lines (right up) and bending/stretching features of GF-based composite (right down). **g** Plots of relative resistance variation versus applied pressure for GF/PDMS and Fe_3_O_4_@Ti_3_C_2_TX/GF/PDMS composites [146] Copyright © 2020 Elsevier B.V

Transparency is also important for next-generation flexible shielding agents because of the demand for visualizations of electronic devices. Among the wide choice of materials, silver nanowires or nanofibers can well satisfy the industrial requirement of sheet resistance < 100 Ω sq^−1^ and transmittance > 90% [141]. Moreover, AgNWs are more chemically stable than other metals like Cu. When compared to carbon-based NWs/NFs, AgNWs could create the networks with higher conductivity. The ferro-ferric oxide (Fe_3_O_4_)-modified AgNW films obtained by Jiao et al*.* [142] exhibited EMI SE of 24.9 dB and transparency of 90%. Due to the high permeability of Fe_3_O_4_, the absorption loss of electromagnetic radiation was improved. By improving the conductivity of silver nanowire film, the shielding effectiveness of silver nanowire EMI shielding film was enhanced. Subsequently, Lei et al. [143] prepared silver nanofiber film *by* a room-temperature roll-to-roll production method, exhibiting superior EMI shielding ability (76 dB, at 100 μm) and great light transmittance (89%, at 1 μm) (Fig. 15a-b). By controlling the fibers diameter and spinning time, the optical transmittance of silver nanofiber film can be varied (Fig. 15c). Furthermore, the film flexibility and bending stability was investigated. Figure 15d shows the test results for flexibility and durability of silver nanofibers and silver nanofiber film. No breaking was observed in silver nanofiber film despite the 180-degree bending.

The rapid developing industry of flexible electronics requires various sensors with high sensitivity and a wide range of responses [144]. Zhao et al*.* [145] prepared the poly(ether-block-amide)/graphene films. With 8.91 vol% graphene, the composite film could reach 30.7 dB of average EMI SE. More interestingly, the poly(ether-block-amide)/graphene film exhibited an almost linear pressure sensing behavior as the external pressure stimulation increased owing to the formation of more conductive paths via decreased distance between adjacent graphene. Also, Nguyen et al*.* [146] demonstrated a multifunctional EMI shielding skin containing freestanding graphene-reinforced PDMS foam decorated by Fe_3_O_4_ NPs-interbedded Ti_3_C_2_T_X_ nanosheets (Fig. 15e), exhibiting remarkable EMI SE of 80 dB in X-band and 77 dB in Ka-band because of high *SE*_*A*_. Besides, it also played a part as a pressure sensor owing to the high electrical conductivity, good elasticity and rapid recovery. It is distinct in Fig. 15f that the sensing material is highly bendable and stretchable. The relative resistance variation (*R* − *R*_0_)/*R*_0_ for the composites is shown in Fig. 15g under varied pressure from 62.4 to 998.9 kPa. The lightweight, highly conductive and flexible composite with favorable response to external pressure is holding great potential for multifunctional EMI shielding skin toward wearable electronics.

Many studies have also shown that flexible EMI shielding materials can demonstrate multiple functions simultaneously. Wang et al*.* [147] recently reported a multifunctional nylon/graphene nanoplatelet (GNP) paper material made of commercial nylon gauze and GNP, via a feasible and scaled method combing vacuum filtration and compression molding. It is investigated that the as-prepared composite paper possesses the advantages of good flexibility and multifunctional properties. When adding 11.8 wt% GNPs, the three-layer composite nylon/GNP paper with a thickness of ~ 180 μm demonstrates both high electrical conductivity and high thermal conductivity of 24.3 S cm^−1^ and15.8 W m^−1^ K^−1^, respectively. In particular, it shows a large EMI SE of 58.1 dB in x-band (8.2–12.4 GHz). Remarkably, the hydrophobicity and flame retardancy of the composites are improved obviously, and the mechanical properties are also satisfactory. For future electronic equipment, the electromagnetic pollution is not the only concern, while flame retardant, heat insulation, water resistance and other features may also be required. Besides, the flexible EMI shielding materials may be required to become optically transparent, electrically conductive, thermally conductive or even sensitive. Therefore, multifunctional integration will be one of the directions in developing flexible EMI shielding materials in the future.

## Conclusions and Prospect

Given the rapid advancement of 5G-related microelectronics and flexible electronics industries, the overwhelmingly generated electromagnetic radiation has definitely become a serious pollution source. Great advances have been achieved in exploration of reliable EMI shielding agents which could reduce or even eliminate detrimental electromagnetic radiation. In this regard, this review describes recent developments of flexible shielding agents based on a comprehensive elaboration of EMI shielding mechanisms, the correlation between absorption and shielding, and lays a specific emphasis on flexible EMI shielding materials with excellent structural integrity and various functional construction. Through literature survey, generally, flexible EMI shielding materials could be obtained via either direct or indirect constructing routes. In terms of the direct route, conductive foam, sponge or aerogel 3D structure present some representative merits and is regarded as excellent contenders. In addition to contributing low weight, the pores in foam, sponge or aerogel promote absorption of EMW energy by multiple scattering at the interfaces within pores. Therefore, foam, sponge or aerogel-structured materials are specialized in flexible portable electronics and defense wearable devices. The indirectly constructed flexible EMI shielding composite materials could show not only great EMI shielding performance but also various excellent properties by introducing nanofillers. On the one hand, the abundant heterogeneous interfaces enhance the interface polarization/relaxation, resulting in magnified interface polarization and multiple reflection-induced *SE*_*A*_. On the other hand, introducing magnetic species into dielectric system could achieve greatly enhanced magnetic loss [148–151]. Meanwhile, the composite materials integrating both magnetic and dielectric components could display both high magnetic loss and dielectric loss [152–155], along with the improved impedance matching [156–159]. From the perspective of flexible materials, carbon-based materials, MXenes and polymers are three mainstream matrixes for constructing the intrinsically flexible substrates or their composites based on nearly most of reported literature.

For carbon-based materials, 1D CNTs and CNFs, and 2D graphene nanosheets could show highly efficient EMW absorption and EMI shielding performance, but their excellent flexibility and mechanical properties can no longer meet the current development of devices. As mentioned previously, various 3D carbon structures including foam, sponge, aerogel are encouraging. Besides the large surface area and highly porous structure, which are conducive to enhanced multiple reflections, the electromagnetic parameters can be adapted by mechanical compression of the foam or sponge. Despite that carbon-based porous microstructures and their composites have been verified to be beneficial toward improved multiple reflections, the underlying mechanism remains unclear. What is the optimal pore size range for EMI shielding? What is the relation between wavelength of incident EMW and aperture? How to evaluate the contribution from multiple reflections and scattering? All these questions need systematic study and urgent breakthrough.

The research on MXene-related materials has been deepened with extensive in-depth investigation from all over the world. MXene exhibits great potential in EMI shielding owing to its laminated structure and high conductivity. By atomic layer clipping and hybridization, the interior conductive networks of MXenes could be regulated to afford high SE and SSE. Nonetheless, the irreversible oxidation of MXene may destroy its microstructure and restrict its EMI SE. Next, it is essential to optimize the hybridization approach to avoid undesired damage of the microstructure for efficient regulation of the network structure. Furthermore, even though the manufacturing method for MXene-based material is coming-of-age at present, in order to achieve large-scale production of MXene, more reliable, safer and economical manufacturing process is admirable.

Polymers are favorable for constructing flexible shielding agents because of excellent elastic–plastic properties. Through addition of nanofillers with high conductivity (CNTs/graphene/CNFs/metal and other fillers) into the polymers, the electrical conductivity and mechanical strength of the resultant composites are further improved. However, dispersion of nanofillers in polymers is a challenging work as van der Waals attraction among carbonaceous fillers would cause agglomerations in the polymers. Therefore, addressing the interface interaction issue between these fillers and polymer matrix is crucial. Besides, some fibrous fillers, such as CNTs and CNFs, present a long structure, but they may transform into curved ones because of ductility during the mixing process, which further increases viscosity. To alleviate these problems, appropriate blending process is also needed along with surface treatment. To sum up, surface treatment and better mixing method of filler are very important to obtain a good EMI shielding for polymer-based composites. The improved wettability of the filler reduces the matrix viscosity, requiring smaller shear stress for filler decentralization in the matrix. This creates multiple contacts between the entangled structure, and the filler in the matrix creates more paths that facilitate electrons to move efficiently through the insulating polymer. Nevertheless, appropriate mixing mechanisms and equipment is challenging in order to achieve such a dispersion in mass production. As described in the literature, it is a challenging to improve structure performance with superior EMI shielding effect under the premise of low quality and low cost.

Based on the above discussion, some prospects concerning the flexible EMI shielding materials are proposed and summarized as follows:

(i) Exploring more novel materials for flexible EMI shielding. Besides the materials discussed above, there are some other emerging materials which are promising for flexible shielding applications. For example, the atomic sheets of boron, called borophene, have shown even more favorable electrical and mechanical properties than graphene [160]. Calculations reveal that corrugated borophene could conduct electricity more easily along the ridge direction than across them, and is also stiffer in this direction than graphene, which suggest its great promise as a new EMI shielding material. What is more, phosphorene, liquid metallic/ionic foams are also holding great promise as future flexible EMI shielding [161].
(ii) Controllable fabrication. The adjustable fabrication and optimization of EMI shielding constituent with designed structure are fundamental for developing the flexible and efficient shielding material. Currently, it is difficult to obtain stable and reproducible materials with precisely controlled morphology, porous structure and multiple interfaces, especially for those sophisticated multilayer or 3D structures such as hydrogels, aerogels and foams that hold great potential for next-generation *SE*_*A*_-dominant shielding materials and devices. More importantly, from an industrial viewpoint, the fabrication of typical shielding materials such as MXene is not environmentally friendly and cost-effective because it usually requires longtime hydrofluoric acid etching, which is quite energy-consuming, time-consuming and dangerous to workers. Meanwhile, the complicated fabrication details, along with unclear synthetic mechanisms have also hindered the scaling up of production of current EMI shielding materials toward practical applications and commercial usage.
(iii) In-depth understanding of shielding mechanisms for "green EMI shielding." The fundamental insights of the composition–structure–property relationship are vital for further optimization of SE. This is the prerequisite for developing highly efficient flexible shielding materials. Currently, the mechanisms of EMI shielding still need to be further studied. Particularly, the future trend of EMI shielding materials is low *SE*_*R*_ and high *SE*_*A*_, *i.e.*, "green EMI shielding." Most of the current EMI shielding materials are dominated by reflection, which comes from high electrical conductivity, while such high reflection will result in secondary pollution of electromagnetic radiation. However, it is still an arduous task to search for more high-absorption and low-reflection materials and develop absorption-dominated EMI shielding mechanisms. In this regard, some advanced techniques such as electron holography could be helpful for revealing the magnetism-related interfacial phenomena toward enhanced "green EMI shielding" performance [162, 163]. On the other hand, it is difficult to implement the absorption, conversion and storage of electromagnetic energy simultaneously to realize the energy recycling. To date, there are still few reports on this meaningful topic. Thus, MXene as an emerging type of materials which presents amazing performance as a variety of catalysts and supercapacitor electrodes [164], may bring about some breakthroughs in the near future.
(iv) Toward multifunctional and practical applications. Till now, highly efficient flexible shielding materials have been extensively studied. The multifunctional level has also been constantly extended from TC and hydrophobicity to transparency, sensing even multiple functions. However, these materials were mostly tested at the laboratory level, while there is still a large gap to practical production. In this regard, the cost control and standardization are two most important prerequisites for scale-up of the production. For cost control, both the selection of EMI flexible shielding material and the consequent processing, including design, fabrication and optimization of multifunctional properties need to be considered more economically. For example, as reported high-performance material for flexible EMI shielding, MXenes are quite expensive, and their fabrication processes are usually quite complicated, which need to be fulfilled at extremely harsh, energy-consuming and time-consuming conditions. One possible way to solve the problems is to reduce the concentration of electrical or magnetic conductive filler in a well-designed 3D scaffold. It could significantly reduce the using amount of MXene, bringing about more cost-effective, low-density flexible shielding materials. Moreover, the controlled porosity can help disperse the conductive species selectively and thereby optimize the generation of conductive network at a lower MXene amount to achieve superior shielding performance. In another concerned respect, for standardization, it is of great significance as well for multifunctional and practical usage of the current laboratory-level shielding materials because it can ensure the stability and reliability with regard to both the production and usage of the materials and devices.

We believe that there is huge space for further development of flexible EMI shielding materials, and this review offers some guidelines for future research on construction of next-generation flexible and multifunctional EMI shielding materials with high performance.

## Abbreviations

g_s_: Green shielding index
EMI: Electromagnetic interference
WLAN: Wireless local area network
IARC: International Agency for Research on Cancer
WHO: World Health Organization
3GPP: 3Rd Generation Partnership Project
EMW: Electromagnetic wave
SE: Shielding efficiency
SE_A_: Absorption loss
SE_R_: Reflection loss
SE_M_: Multiple reflection loss
SE_T_: The total SE of EMI
3D: Three-dimensional
CNT: Carbon nanotube
GO: Graphene oxide
rGO: Reduced graphene oxide
G-film: Graphene film
G-foam: Graphene foam
CVD: Chemical vapor deposition
SSE: Specific shielding efficiency
2D: Two-dimensional
1D: One-dimensional
PDMS: POLYDIMETHYLSILOXANE
CNFs: Carbon nanofibers
CB: Carbon black
NPs: Nanoparticles
ABS: Acrylonitrile–butadiene–styrene
AgNW: Silver nanowire
PVA: Polyvinyl alcohol
SCF: Short carbon fiber
EVA: Vinyl acetate
PAN: Polyacrylonitrile
PDA: Polydopamine
PANI: Polyaniline
PF: Polyfuran
PTH: Polythiophene
PPy: Polypyrrole
PEDOT: Poly(3,4-ethylenedioxythiophene)
PPP: Polyparaphenylene
PA: Polyacetylene
PPV: Poly(p-phenylene vinylene)
PSS: Poly(styrenesulfonate)
PIPD: Poly(pyridobisimidazole)
PDDA: Polydimethyl diallyl ammonium
TC: Thermal conductivity
CPFCs: Conductive polymer fabric composites
PET: Poly(ethylene terephthalate

## References

[1] T. Alsop, Number of wireless local area network (WLAN) connected devices worldwide from 2016 to 2021. (2020). https://www.statista.com/statistics/802706/world-wlan-connected-device/

[2] Baan R, Grosse Y, Lauby-Secretan B, Ghissassi FE, Bouvard V (2011). Carcinogenicity of radiofrequency electromagnetic fields. Lancet Oncol..

[3] Danker-Hopfe H, Dorn H, Bolz T, Peter A, Hansen ML (2016). Effects of mobile phone exposure (GSM 900 and WCDMA/UMTS) on polysomnography based sleep quality: an intra- and inter-individual perspective. Environ. Res..

[4] Liu L, Deng H, Tang X, Lu Y, Zhou J (2021). Specific electromagnetic radiation in the wireless signal range increases wakefulness in mice. PNAS.

[5] Shahin S, Banerjee S, Swarup V, Singh SP, Chaturvedi CM (2018). From the cover: 2. 45-GHz microwave radiation impairs hippocampal learning and spatial memory: involvement of local stress mechanism-induced suppression of iGluR/ERK/CREB signaling. Toxicol. Sci..

[6] Bogers R, Gils AV, Clahsen S, Vercruijsse W, Kamp IV (2018). Individual variation in temporal relationships between exposure to radiofrequency electromagnetic fields and non-specific physical symptoms: a new approach in studying ‘electrosensitivity’. Environ. Int..

[7] Falcioni L, Bua L, Tibaldi E, Lauriola M, Angelis LD (2018). Report of final results regarding brain and heart tumors in sprague-dawley rats exposed from prenatal life until natural death to mobile phone radiofrequency field representative of a 1.8 GHz GSM base station environmental emission. Environ. Res..

[8] Smith-Roe SL, Wyde ME, Stout MD, Winters JW, Hobbs CA (2020). Evaluation of the genotoxicity of cell phone radiofrequency radiation in male and female rats and mice following subchronic exposure. Environ. Mol. Mutagen..

[9] The 3GPP specification. https://www.3gpp.org/DynaReport/38-series.htm

[10] Han Y, Liu Y, Han L, Lin J, Jin P (2017). High-performance hierarchical graphene/metal-mesh film for optically transparent electromagnetic interference shielding. Carbon.

[11] Chen Z, Xu C, Ma C, Ren W, Cheng HM (2013). Lightweight and flexible graphene foam composites for high-performance electromagnetic interference shielding. Adv. Mater..

[12] Zhang Y, Qiu M, Yu Y, Wen B, Cheng L (2017). A novel polyaniline-coated bagasse fiber composite with core–shell heterostructure provides effective electromagnetic shielding performance. ACS Appl. Mater. Interfaces.

[13] Song WL, Cao MS, Lu MM, Bi S, Wang CY (2014). Flexible graphene/polymer composite films in sandwich structures for effective electromagnetic interference shielding. Carbon.

[14] Song WL, Guan XT, Fan LZ, Cao WQ, Wang CY (2015). Tuning three-dimensional textures with graphene aerogels for ultra-light flexible graphene/texture composites of effective electromagnetic shielding. Carbon.

[15] Tan YJ, Li J, Gao Y, Li J, Guo S (2018). A facile approach to fabricating silver-coated cotton fiber non-woven fabrics for ultrahigh electromagnetic interference shielding. Appl. Surf. Sci..

[16] Watts CM, Liu X, Padilla WJ (2012). Metamaterial electromagnetic wave absorbers. Adv. Mater..

[17] Zhou Q, Yin X, Ye F, Mo R, Tang Z (2019). Optically transparent and flexible broadband microwave metamaterial absorber with sandwich structure. J. Appl. Phys. A.

[18] Han M, Yin X, Hantanasirisakul K, Li X, Iqbal A (2019). Anisotropic MXene aerogels with a mechanically tunable ratio of electromagnetic wave reflection to absorption. Adv. Opt. Mater..

[19] Ghosh S, Ganguly S, Remanan S, Mondal S, Jana S (2018). Ultra-light weight, water durable and flexible highly electrical conductive polyurethane foam for superior electromagnetic interference shielding materials. J. Mater. Sci. Mater. Electron..

[20] Chen M, Zhang L, Duan S, Jing S, Jiang H (2014). Highly conductive and flexible polymer composites with improved mechanical and electromagnetic interference shielding performances. Nanoscale.

[21] Hsiao ST, Ma CCM, Liao WH, Wang YS, Li SM (2014). Lightweight and flexible reduced graphene oxide/water-borne polyurethane composites with high electrical conductivity and excellent electromagnetic interference shielding performance. ACS Appl. Mater. Interfaces.

[22] Cui C, Xiang C, Geng L, Lai X, Guo R (2019). Flexible and ultrathin electrospun regenerate cellulose nanofibers and d-Ti_3_C_2_T_x_ (MXene) composite film for electromagnetic interference shielding. J. Alloys Compd..

[23] Song WL, Wang J, Fan LZ, Li Y, Wang CY (2014). Interfacial engineering of carbon nanofiber–graphene–carbon nanofiber heterojunctions in flexible lightweight electromagnetic shielding networks. ACS Appl. Mater. Interfaces.

[24] Li R, Lin H, Lan P, Gao J, Huang Y (2018). Lightweight cellulose/carbon fiber composite foam for electromagnetic interference (EMI) shielding. Polymers.

[25] Sahu KR, De U (2018). Polymer composites for flexible electromagnetic shields. Macromol. Symp..

[26] Wang Y, Gu FQ, Ni LJ, Liang K, Marcus K (2017). Easily fabricated and lightweight PPy/PDA/AgNW composites for excellent electromagnetic interference shielding. Nanoscale.

[27] Sushmita K, Madras G, Bose S (2020). Polymer nanocomposites containing semiconductors as advanced materials for EMI shielding. ACS Omega.

[28] Cao MS, Cai YZ, He P, Shu JC, Cao WQ (2019). 2D MXenes: electromagnetic property for microwave absorption and electromagnetic interference shielding. Chem. Eng. J..

[29] Zhang DQ, Liu TT, Shu JC, Liang S, Wang XX (2019). Self-assembly construction of WS_2_–rGO architecture with green EMI shielding. ACS Appl. Mater. Interfaces.

[30] Zhang D, Liu T, Cheng J, Chai J, Yang X (2019). Light-weight and low-cost electromagnetic wave absorbers with high performances based on biomass-derived reduced graphene oxides. Nanotechnology.

[31] Zhang D, Liang S, Chai J, Liu T, Yang X (2019). Highly effective shielding of electromagnetic waves in MoS_2_ nanosheets synthesized by a hydrothermal method. J. Phys. Chem. Solids..

[32] Zhang D, Yang X, Cheng J, Lu M, Zhao B (2013). Facile preparation, characterization, and highly effective microwave absorption performance of CNTs/Fe_3_O_4_/PANI nanocomposites. J. Nanomater..

[33] Zhang D, Cheng J, Yang X, Zhao B, Cao M (2014). Electromagnetic and microwave absorbing properties of magnetite nanoparticles decorated carbon nanotubes/polyaniline multiphase heterostructures. J. Mater. Sci..

[34] Chai J, Cheng J, Zhang D, Xiong Y, Yang X (2020). Enhancing electromagnetic wave absorption performance of Co_3_O_4_ nanoparticles functionalized MoS_2_ nanosheets. J. Alloys Compd..

[35] Xiong R, Hu K, Grant AM, Ma R, Xu W (2016). Ultrarobust transparent cellulose nanocrystal-graphene membranes with high electrical conductivity. Adv. Opt. Mater..

[36] Iqbal A, Sambyal P, Koo CM (2020). 2D MXenes for electromagnetic shielding: a review. Adv. Funct. Mater..

[37] Zhang H, Liu T, Huang Z, Cheng J, Wang H (2021). Engineering flexible and green electromagnetic interference shielding materials with high performance through modulating WS_2_ nanosheets on carbon fibers. J. Materiomics..

[38] Zhang D, Xiong Y, Cheng J, Chai J, Liu T (2020). Synergetic dielectric loss and magnetic loss towards superior microwave absorption through hybridization of few-layer WS_2_ nanosheets with NiO nanoparticles. Sci. Bull..

[39] Zhang D, Chai J, Cheng J, Jia Y, Yang X (2018). Highly efficient microwave absorption properties and broadened absorption bandwidth of MoS_2_-iron oxide hybrids and MoS_2_-based reduced graphene oxide hybrids with hetero-structures. Appl. Surf. Sci..

[40] Cheng J, Zhang H, Xiong Y, Gao L, Wen B (2021). Construction of multiple interfaces and dielectric/magnetic heterostructures in electromagnetic wave absorbers with enhanced absorption performance: a review. J. Materiomics.

[41] Al-Saleh MH, Saadeh WH, Sundararaj U (2013). EMI shielding effectiveness of carbon based nanostructured polymeric materials: a comparative study. Carbon.

[42] Kwon S, Ma R, Kim U, Choi HR, Baik S (2014). Flexible electromagnetic interference shields made of silver flakes, carbon nanotubes and nitrile butadiene rubber. Carbon.

[43] Al-Saleh MH, Sundararaj U (2009). Electromagnetic interference shielding mechanisms of CNT/polymer composites. Carbon.

[44] Song WL, Cao MS, Fan LZ, Lu MM, Li Y (2014). Highly ordered porous carbon/wax composites for effective electromagnetic attenuation and shielding. Carbon.

[45] Lin S, Liu J, Wang Q, Zu D, Wang H (2020). Highly robust, flexible, and large-scale 3D-metallized sponge for high-performance electromagnetic interference shielding. Adv. Mater. Technol..

[46] Liu X, Yu Z, Ishikawa R, Chen L, Liu X (2017). Single-source-precursor derived rGO/CNTs-SiCN ceramic nanocomposite with ultra-high electromagnetic shielding effectiveness. Acta Mater..

[47] Han M, Yin X, Li X, Anasori B, Zhang L (2017). Laminated and two-dimensional carbon-supported microwave absorbers derived from MXenes. ACS Appl. Mater. Interfaces.

[48] Yan DX, Pang H, Li B, Vajtai R, Xu L (2015). Structured reduced graphene oxide/polymer composites for ultra-efficient electromagnetic interference shielding. Adv. Funct. Mater..

[49] Zhou E, Xi J, Guo Y, Liu Y, Xu Z (2018). Synergistic effect of graphene and carbon nanotube for high-performance electromagnetic interference shielding films. Carbon.

[50] Gupta TK, Singh BP, Mathur RB, Dhakate SR (2014). Multi-walled carbon nanotube–graphene–polyaniline multiphase nanocomposite with superior electromagnetic shielding effectiveness. Nanoscale.

[51] Hong Y, Lee C, Jeong C, Lee D, Kim K (2003). Method and apparatus to measure electromagnetic interference shielding efficiency and its shielding characteristics in broadband frequency ranges. Rev. Sci. Instrum..

[52] Singh AP, Garg P, Alam F, Singh K, Mathur RB (2012). Phenolic resin-based composite sheets filled with mixtures of reduced graphene oxide, γ-Fe_2_O_3_ and carbon fibers for excellent electromagnetic interference shielding in the X-band. Carbon.

[53] Lu S, Shao J, Ma K, Chen D, Wang X (2018). Flexible, mechanically resilient carbon nanotube composite films for high-efficiency electromagnetic interference shielding. Carbon.

[54] Li N, Huang Y, Du F, He X, Lin X (2006). Electromagnetic interference (EMI) shielding of single-walled carbon nanotube epoxy composites. Nano Lett..

[55] Bian XM, Liu L, Li HB, Wang CY, Xie Q (2016). Construction of three-dimensional graphene interfaces into carbon fiber textiles for increasing deposition of nickel nanoparticles: flexible hierarchical magnetic textile composites for strong electromagnetic shielding. Nanotechnology.

[56] Li Y, Wang B, Sui X, Xu H, Zhang L (2017). Facile synthesis of microfibrillated cellulose/organosilicon/polydopamine composite sponges with flame retardant properties. Cellulose.

[57] Wang B, Li W, Deng J (2017). Chiral 3D porous hybrid foams constructed by graphene and helically substituted polyacetylene: preparation and application in enantioselective crystallization. J. Mater. Sci..

[58] Shahzad F, Alhabeb M, Hatter CB, Anasori B, Hong SM (2016). Electromagnetic interference shielding with 2D transition metal carbides (MXenes). Science.

[59] Naguib M, Mochalin VN, Barsoum MW, Gogotsi Y (2014). 25th anniversary article: MXenes: a new family of two-dimensional materials. Adv. Mater..

[60] Xu H, Yin X, Li X, Li M, Liang S (2019). Lightweight Ti_2_CTx MXene/poly(vinyl alcohol) composite foams for electromagnetic wave shielding with absorption-dominated feature. ACS Appl. Mater. Interfaces.

[61] Zhang D, Liu T, Zhang M, Zhang H, Yang X (2020). Confinedly growing and tailoring of Co_3_O_4_ clusters-WS_2_ nanosheets for highly efficient microwave absorption. Nanotechnology.

[62] Qiang R, Du Y, Zhao H, Wang Y, Tian C (2015). Metal organic framework-derived Fe/C nanocubes toward efficient microwave absorption. J. Mater. Chem. A.

[63] Du Y, Liu W, Qiang R, Wang Y, Han X (2014). Shell thickness-dependent microwave absorption of core–shell Fe_3_O_4_@C composites. ACS Appl. Mater. Interfaces.

[64] Liu B, Cheng J, Peng HQ, Chen D, Cui X (2019). In situ nitridated porous nanosheet networked Co_3_O_4_–Co_4_N heteronanostructures supported on hydrophilic carbon cloth for highly efficient electrochemical hydrogen evolution. J. Mater. Chem. A.

[65] Wang H, Dai Y, Gong W, Geng D, Ma S (2013). Broadband microwave absorption of CoNi@C nanocapsules enhanced by dual dielectric relaxation and multiple magnetic resonances. Appl. Phys. Lett..

[66] Song WL, Cao MS, Hou ZL, Fang XY, Shi XL (2009). High dielectric loss and its monotonic dependence of conducting-dominated multiwalled carbon nanotubes/silica nanocomposite on temperature ranging from 373 to 873 K in X-band. Appl. Phys. Lett..

[67] Song WL, Cao M, Hou Z, Yuan J, Fang X (2009). High-temperature microwave absorption and evolutionary behavior of multiwalled carbon nanotube nanocomposite. Scr. Mater..

[68] Sun G, Dong B, Cao M, Wei B, Hu C (2011). Hierarchical dendrite-like magnetic materials of Fe_3_O_4_, γ-Fe_2_O_3_, and Fe with high performance of microwave absorption. Chem. Mater..

[69] Tong G, Wu W, Guan J, Qian H, Yuan J (2011). Synthesis and characterization of nanosized urchin-like α-Fe_2_O_3_ and Fe_3_O_4_: microwave electromagnetic and absorbing properties. J. Alloys Compd..

[70] Wang L, Huang Y, Sun X, Huang H, Liu P (2014). Synthesis and microwave absorption enhancement of graphene@Fe_3_O_4_@SiO_2_@NiO nanosheet hierarchical structures. Nanoscale.

[71] Zhang Y, Wang X, Cao M (2018). Confinedly implanted NiFe_2_O_4_-rGO: cluster tailoring and highly tunable electromagnetic properties for selective-frequency microwave absorption. Nano Res..

[72] Zhang D, Cheng J, Chai J, Deng J, Ren R (2018). Magnetic-field-induced dielectric behaviors and magneto-electrical coupling of multiferroic compounds containing cobalt ferrite/barium calcium titanate composite fibers. J. Alloys Compd..

[73] Zhang Y, Huang Y, Zhang T, Chang H, Xiao P (2015). Broadband and tunable high-performance microwave absorption of an ultralight and highly compressible graphene foam. Adv. Mater..

[74] Cheng Y, Zhao H, Lv H, Shi T, Ji G (2020). Lightweight and flexible cotton aerogel composites for electromagnetic absorption and shielding applications. Adv. Electron. Mater..

[75] Cheng Y, Hu P, Zhou S, Yan L, Sun B (2018). Achieving tunability of effective electromagnetic wave absorption between the whole X-band and Ku-band via adjusting PPy loading in SiC nanowires/graphene hybrid foam. Carbon.

[76] Zhou C, Geng S, Xu X, Wang T, Zhang L (2016). Lightweight hollow carbon nanospheres with tunable sizes towards enhancement in microwave absorption. Carbon.

[77] Han M, Yin X, Wu H, Hou Z, Song C (2016). Ti_3_C_2_ MXenes with modified surface for high-performance electromagnetic absorption and shielding in the X-band. ACS Appl. Mater. Interfaces.

[78] Zhou C, Wu C, Yan M (2019). A versatile strategy towards magnetic/dielectric porous heterostructure with confinement effect for lightweight and broadband electromagnetic wave absorption. Chem. Eng. J..

[79] Peng M, Qin F (2021). Clarification of basic concepts for electromagnetic interference shielding effectiveness. J. Appl. Phys..

[80] Yu Z, Dai T, Yuan S, Zou H, Liu P (2020). Electromagnetic interference shielding performance of anisotropic polyimide/graphene composite aerogels. ACS Appl. Mater. Interfaces.

[81] Zhang B, Wang J, Peng J, Sun J, Su X (2019). Double-shell PANS@PANI@Ag hollow microspheres and graphene dispersed in epoxy with enhanced microwave absorption. J. Mater. Sci. Mater. Electron..

[82] Lu B, Dong XL, Huang H, Zhang XF, Zhu XG (2008). Microwave absorption properties of the core/shell-type iron and nickel nanoparticles. J. Magn. Magn. Mater..

[83] Zhao S, Gao Z, Chen C, Wang G, Zhang B (2016). Alternate nonmagnetic and magnetic multilayer nanofilms deposited on carbon nanocoils by atomic layer deposition to tune microwave absorption property. Carbon.

[84] Zhao S, Yan L, Tian X, Liu Y, Chen C (2018). Flexible design of gradient multilayer nanofilms coated on carbon nanofibers by atomic layer deposition for enhanced microwave absorption performance. Nano Res..

[85] Liu J, You W, Yu J, Liu X, Zhang X (2019). Electron holography of yolk–shell Fe_3_O_4_@mSiO_2_ microspheres for use in microwave absorption. ACS Appl. Nano Mater..

[86] Yu M, Liang C, Liu M, Liu X, Yuan K (2014). Yolk–shell Fe_3_O_4_@ZrO_2_ prepared by a tunable polymer surfactant assisted sol–gel method for high temperature stable microwave absorption. J. Mater. Chem. C.

[87] Cheng Y, Li Z, Li Y, Dai S, Ji G (2018). Rationally regulating complex dielectric parameters of mesoporous carbon hollow spheres to carry out efficient microwave absorption. Carbon.

[88] Deng Y, Zhao L, Shen B, Liu L, Hu W (2006). Microwave characterization of submicrometer-sized nickel hollow sphere composites. J. Appl. Phys..

[89] Xu H, Yin X, Zhu M, Li M, Zhang H (2019). Constructing hollow graphene nano-spheres confined in porous amorphous carbon particles for achieving full X band microwave absorption. Carbon.

[90] Luo J, Zhang K, Cheng M, Gu M, Sun X (2020). MoS_2_ spheres decorated on hollow porous ZnO microspheres with strong wideband microwave absorption. Chem. Eng. J..

[91] Xu H, Yin X, Zhu M, Han M, Hou Z (2017). Carbon hollow microspheres with a designable mesoporous shell for high-performance electromagnetic wave absorption. ACS Appl. Mater. Interfaces.

[92] Lv H, Ji G, Liu W, Zhang H, Du Y (2015). Achieving hierarchical hollow carbon@Fe@Fe_3_O_4_ nanospheres with superior microwave absorption properties and lightweight features. J. Mater. Chem. C.

[93] Hou J, Zhang L, Qiu H, Duan W, Wang X (2017). Fabrication and microwave absorption performances of hollow-structure Fe_3_O_4_/PANI microspheres. J. Mater. Sci. Mater. Electron..

[94] Raagulan K, Kim BM, Chai KY (2020). Recent advancement of electromagnetic interference (EMI) shielding of two dimensional (2D) MXene and graphene aerogel composites. Nanomaterials.

[95] Bi S, Zhang L, Mu C, Liu M, Hu X (2017). Electromagnetic interference shielding properties and mechanisms of chemically reduced graphene aerogels. Appl. Surf. Sci..

[96] Kong L, Yin X, Zhang Y, Yuan X, Li Q (2013). Electromagnetic wave absorption properties of reduced graphene oxide modified by maghemite colloidal nanoparticle clusters. J. Phys. Chem. C.

[97] González M, Baselga J, Pozuelo J (2019). Modulating the electromagnetic shielding mechanisms by thermal treatment of high porosity graphene aerogels. Carbon.

[98] Chen Y, Zhang H, Zeng G (2018). Tunable and high performance electromagnetic absorber based on ultralight 3D graphene foams with aligned structure. Carbon.

[99] Ma Z, Kang S, Ma J, Shao L, Zhang Y (2020). Ultraflexible and mechanically strong double-layered aramid nanofiber–Ti_3_C_2_Tx MXene/silver nanowire nanocomposite papers for high-performance electromagnetic interference shielding. ACS Nano.

[100] Shen B, Li Y, Yi D, Zhai W, Wei X (2016). Microcellular graphene foam for improved broadband electromagnetic interference shielding. Carbon.

[101] Crespo M, González M, Elías AL, Rajukumar LP, Baselga J (2014). Ultra-light carbon nanotube sponge as an efficient electromagnetic shielding material in the GHz range. Phys. Status Solidi RRL.

[102] Bian R, Lin R, Wang G, Lu G, Zhi W (2018). 3D assembly of Ti_3_C_2_-MXene directed by water/oil interfaces. Nanoscale.

[103] Shi S, Qian B, Wu X, Sun H, Wang H (2019). Self-assembly of MXene-surfactants at liquid–liquid interfaces: From structured liquids to 3D aerogels. Angew. Chem. Int. Ed..

[104] Shang T, Lin Z, Qi C, Liu X, Li P (2019). 3D macroscopic architectures from self-assembled MXene hydrogels. Adv. Funct. Mater..

[105] Liu J, Zhang HB, Sun R, Liu Y, Liu Z (2017). Hydrophobic, flexible, and lightweight MXene foams for high-performance electromagnetic-interference shielding. Adv. Mater..

[106] Qian K, Zhou Q, Wu H, Fang J, Miao M (2021). Carbonized cellulose microsphere@void@MXene composite films with egg-box structure for electromagnetic interference shielding. Compos. A Appl. Sci. Manuf..

[107] Hatchett DW, Josowicz M (2008). Composites of intrinsically conducting polymers as sensing nanomaterials. Chem. Rev..

[108] Liu X, Zhang L, Yin X, Ye F, Liu Y (2016). Flexible thin SiC fiber fabrics using carbon nanotube modification for improving electromagnetic shielding properties. Mater. Des..

[109] Zhu S, Xing C, Wu F, Zuo X, Zhang Y (2019). Cake-like flexible carbon nanotubes/graphene composite prepared via a facile method for high-performance electromagnetic interference shielding. Carbon.

[110] Mondal S, Ganguly S, Das P, Khastgir D, Das NC (2017). Low percolation threshold and electromagnetic shielding effectiveness of nano-structured carbon based ethylene methyl acrylate nanocomposites. Compos. B Eng..

[111] Tibbetts GG, Lake ML, Strong KL, Rice BP (2007). A review of the fabrication and properties of vapor-grown carbon nanofiber/polymer composites. Compos. Sci. Technol..

[112] Shen B, Li Y, Zhai W, Zheng W (2016). Compressible graphene-coated polymer foams with ultralow density for adjustable electromagnetic interference (EMI) shielding. ACS Appl. Mater. Interfaces.

[113] Jia LC, Li YK, Yan DX (2017). Flexible and efficient electromagnetic interference shielding materials from ground tire rubber. Carbon.

[114] Zhan Y, Oliviero M, Wang J, Sorrentino A, Buonocore GG (2019). Enhancing the EMI shielding of natural rubber-based supercritical CO_2_ foams by exploiting their porous morphology and CNT segregated networks. Nanoscale.

[115] Feng D, Liu P, Wang Q (2019). Exploiting the piezo resistivity and EMI shielding of polyetherimide/carbon nanotube foams by tailoring their porous morphology and segregated CNT networks. Compos. A Appl. Sci. Manuf..

[116] Kong L, Yin X, Xu H, Yuan X, Wang T (2019). Powerful absorbing and lightweight electromagnetic shielding CNTs/rGO composite. Carbon.

[117] Mei H, Zhao X, Xia J, Wei F, Han D (2018). Compacting CNT sponge to achieve larger electromagnetic interference shielding performance. Mater. Des..

[118] Lu D, Mo Z, Liang B, Yang L, He Z (2018). Flexible, lightweight carbon nanotube sponges and composites for high-performance electromagnetic interference shielding. Carbon.

[119] Sun X, Liu X, Shen X, Wu Y, Wang Z (2016). Graphene foam/carbon nanotube/poly (dimethyl siloxane) composites for exceptional microwave shielding. Compos. A Appl. Sci. Manuf..

[120] Wan YJ, Zhu PL, Yu SH, Sun R, Wong CP (2018). Anticorrosive, ultralight, and flexible carbon-wrapped metallic nanowire hybrid sponges for highly efficient electromagnetic interference shielding. Small.

[121] Zhan Z, Song Q, Zhou Z, Lu C (2019). Ultrastrong and conductive MXene/cellulose nanofiber films enhanced by hierarchical nano-architecture and interfacial interaction for flexible electromagnetic interference shielding. J. Mater. Chem. C.

[122] Hu D, Huang X, Li S, Jiang P (2020). Flexible and durable cellulose/MXene nanocomposite paper for efficient electromagnetic interference shielding. Compos. Sci. Technol..

[123] Feng X, Ning J, Wang B, Guo H, Xia M (2020). Functional integrated electromagnetic interference shielding in flexible micro-supercapacitors by cation-intercalation typed Ti_3_C_2_Tx MXene. Nano Energy.

[124] Zhao S, Zhang HB, Luo JQ, Wang QW, Xu B (2018). Highly electrically conductive three-dimensional Ti_3_C_2_T_x_ MXene/reduced graphene oxide hybrid aerogels with excellent electromagnetic interference shielding performances. ACS Nano.

[125] Yousefi N, Sun X, Lin X, Shen X, Jia J (2014). Highly aligned graphene/polymer nanocomposites with excellent dielectric properties for high-performance electromagnetic interference shielding. Adv. Mater..

[126] Yao B, Hong W, Chen T, Han Z, Xu X (2020). Highly stretchable polymer composite with strain-enhanced electromagnetic interference shielding effectiveness. Adv. Mater..

[127] Li P, Du D, Guo L, Guo Y, Ouyang J (2016). Stretchable and conductive polymer films for high-performance electromagnetic interference shielding. J. Mater. Chem. C.

[128] Zhao B, Zeng S, Li X, Guo X, Bai Z (2020). Flexible PVDF/carbon materials/Ni composite films maintaining strong electromagnetic wave shielding under cyclic microwave irradiation. J. Mater. Chem. C.

[129] Das N, Chaki T, Khastgir D, Chakraborty A (2001). Electromagnetic interference shielding effectiveness of ethylene vinyl acetate based conductive composites containing carbon fillers. J. Appl. Polym. Sci..

[130] Cheng J, Yang X, Dong L, Yuan Z, Wang W (2017). Effective nondestructive evaluations on UHMWPE/Recycled-PA6 blends using FTIR imaging and dynamic mechanical analysis. Polym. Test..

[131] Li H, Jensen M, Wang N, Chen Y, Gao Y (2019). CuxS/PAN 3D nanofiber mats as ultra-lightweight and flexible electromagnetic interference shielding materials. Macromol. Mater. Eng..

[132] Zeng Z, Jiang F, Yue Y, Han D, Lin L (2020). Flexible and ultrathin waterproof cellular membranes based on high-conjunction metal-wrapped polymer nanofibers for electromagnetic interference shielding. Adv. Mater..

[133] Shen W, Estevez D, Zhou L, Xu P, Qin F (2022). Stretchable silver@CNT-poly(vinyl alcohol) films with efficient electromagnetic shielding prepared by polydopamine functionalization. Polymer.

[134] Wu Y, Wang Z, Liu X, Shen X, Zheng Q (2017). Ultralight graphene foam/conductive polymer composites for exceptional electromagnetic interference shielding. ACS Appl. Mater. Interfaces.

[135] Chen Z, Ren W, Gao L, Liu B, Pei S (2011). Three-dimensional flexible and conductive interconnected graphene networks grown by chemical vapour deposition. Nat. Mater..

[136] Li J, Liu H, Guo J, Hu Z, Wang Z (2017). Flexible, conductive, porous, fibrillar polymer–gold nanocomposites with enhanced electromagnetic interference shielding and mechanical properties. J. Mater. Chem. C.

[137] Chaudhary A, Teotia S, Kumar R, Gupta V, Dhakate SR (2019). Multi-component framework derived SiC composite paper to support efficient thermal transport and high EMI shielding performance. Compos. B Eng..

[138] Liang C, Ruan K, Zhang Y, Gu J (2020). Multifunctional flexible electromagnetic interference shielding silver nanowires/cellulose films with excellent thermal management and joule heating performances. ACS Appl. Mater. Interfaces.

[139] Luo J, Wang L, Huang X, Li B, Guo Z (2019). Mechanically durable, highly conductive, and anticorrosive composite fabrics with excellent self-cleaning performance for high-efficiency electromagnetic interference shielding. ACS Appl. Mater. Interfaces.

[140] Wang QW, Zhang HB, Liu J, Zhao S, Xie X (2019). Multifunctional and water-resistant MXene-decorated polyester textiles with outstanding electromagnetic interference shielding and joule heating performances. Adv. Funct. Mater..

[141] Chu HC, Chang YC, Lin Y, Chang SH, Chang WC (2016). Spray-deposited large-area copper nanowire transparent conductive electrodes and their uses for touch screen applications. ACS Appl. Mater. Interfaces.

[142] Wang Z, Jiao B, Qing Y, Nan H, Huang L (2020). Flexible and transparent ferroferric oxide-modified silver nanowire film for efficient electromagnetic interference shielding. ACS Appl. Mater. Interfaces.

[143] Lin S, Wang H, Wu F, Wang Q, Bai X (2019). Room-temperature production of silver-nanofiber film for large-area, transparent and flexible surface electromagnetic interference shielding. npj Electron Flex.

[144] Panahi-Sarmad M, Noroozi M, Abrisham M, Eghbalinia S, Teimoury F (2020). A comprehensive review on carbon-based polymer nanocomposite foams as electromagnetic interference shields and piezoresistive sensors. ACS Appl. Electron. Mater..

[145] Zhao B, Zhang X, Deng J, Zhang C, Li Y (2020). Flexible PEBAX/graphene electromagnetic shielding composite films with a negative pressure effect of resistance for pressure sensors applications. RSC Adv..

[146] Nguyen VT, Min BK, Yi Y, Kim SJ, Choi CG (2020). MXene(Ti_3_C_2_Tx)/graphene/PDMS composites for multifunctional broadband electromagnetic interference shielding skins. Chem. Eng. J..

[147] Wang W, Ma X, Shao Y, Qi X, Yang J (2021). Flexible, multifunctional, and thermally conductive nylon/graphene nanoplatelet composite papers with excellent EMI shielding performance, improved hydrophobicity and flame resistance. J. Mater. Chem. A.

[148] Liu Q, Cao Q, Bi H, Liang C, Yuan K (2016). Coni@SiO_2_@TiO_2_ and CoNi@air@TiO_2_ microspheres with strong wideband microwave absorption. Adv. Mater..

[149] Liu Q, Xu X, Xia W, Che R, Chen C (2015). Dependency of magnetic microwave absorption on surface architecture of Co_20_Ni_80_ hierarchical structures studied by electron holography. Nanoscale.

[150] Liu Q, Cao Q, Zhao X, Bi H, Wang C (2015). Insights into size-dominant magnetic microwave absorption properties of CoNi microflowers via off-axis electron holography. ACS Appl. Mater. Interfaces.

[151] Zhang D, Wang H, Cheng J, Han C, Yang X (2020). Conductive WS_2_-NS/CNTs hybrids based 3D ultra-thin mesh electromagnetic wave absorbers with excellent absorption performance. Appl. Surf. Sci..

[152] Huang Z, Cheng J, Zhang H, Xiong Y, Zhou Z (2022). High-performance microwave absorption enabled by Co_3_O_4_ modified VB-group laminated VS_2_ with frequency modulation from S-band to Ku-band. J. Mater. Sci. Technol..

[153] Zhang H, Cheng J, Wang H, Huang Z, Zheng Q (2021). Initiating VB-group laminated NbS2 electromagnetic wave absorber toward superior absorption bandwidth as large as 6 48 GHz through phase engineering modulation. Adv. Funct. Mater..

[154] Ye X, Chen Z, Ai S, Hou B, Zhang J (2019). Porous SiC/melamine-derived carbon foam frameworks with excellent electromagnetic wave absorbing capacity. J. Adv. Ceram..

[155] Sun H, Che R, You X, Jiang Y, Yang Z (2014). Cross-stacking aligned carbon-nanotube films to tune microwave absorption frequencies and increase absorption intensities. Adv. Mater..

[156] Yuan K, Che R, Cao Q, Sun Z, Yue Q (2015). Designed fabrication and characterization of three-dimensionally ordered arrays of core–shell magnetic mesoporous carbon microspheres. ACS Appl. Mater. Interfaces.

[157] Che RC, Peng LM, Duan XF, Chen Q, Liang XL (2004). Microwave absorption enhancement and complex permittivity and permeability of Fe encapsulated within carbon nanotubes. Adv. Mater..

[158] Wu Z, Pei K, Xing L, Yu X, You W (2019). Enhanced microwave absorption performance from magnetic coupling of magnetic nanoparticles suspended within hierarchically tubular composite. Adv. Funct. Mater..

[159] Liu P, Gao S, Zhang G, Huang Y, You W (2021). Hollow engineering to Co@N-doped carbon nanocages via synergistic protecting-etching strategy for ultrahigh microwave absorption. Adv. Funct. Mater..

[160] Mannix AJ, Zhou XF, Kiraly B, Wood JD, Alducin D (2015). Synthesis of borophenes: anisotropic, two-dimensional boron polymorphs. Science.

[161] Zhao B, Hamidinejad M, Wang S, Bai P, Che R (2021). Advances in electromagnetic shielding properties of composite foams. J. Mater. Chem. A.

[162] Wang L, Huang M, Qian X, Liu L, You W (2021). Confined magnetic-dielectric balance boosted electromagnetic wave absorption. Small.

[163] Zhang J, Wang Z, Li J, Dong Y, He A (2022). Magnetic-electric composite coating with oriented segregated structure for enhanced electromagnetic shielding. J. Mater. Sci. Technol..

[164] Li X, Wen C, Yang L, Zhang R, Li X (2021). MXene/FeCo films with distinct and tunable electromagnetic wave absorption by morphology control and magnetic anisotropy. Carbon.

[165] Li CB, Li YJ, Zhao Q, Luo Y, Yang GY (2020). Electromagnetic interference shielding of graphene aerogel with layered microstructure fabricated via mechanical compression. ACS Appl. Mater. Interfaces.

